# Multidrug-resistant sepsis in special newborn care units in five district hospitals in India: a prospective cohort study

**DOI:** 10.1016/S2214-109X(24)00564-3

**Published:** 2025-02-26

**Authors:** Kajal Jain, Vivek Kumar, Nishad Plakkal, Deepak Chawla, Atul Jindal, Reeta Bora, Neeraj Gupta, Apurba Sastry, Nidhi Singla, Anudita Bhargava, Reema Nath, Vijayalakshmi Nag, Sarita Mohapatra, Nitya Wadhwa, Ramesh Agarwal, M Jeeva Sankar, DH study collaborators, Kajal Jain, Vivek Kumar, Nishad Plakkal, Deepak Chawla, Atul Jindal, Reeta Bora, Neeraj Gupta, Apurba Sastry, Nidhi Singla, Anudita Bhargava, Reema Nath, Vijaya Lakshmi Nag, Sarita Mohapatra, Nitya Wadhwa, Ramesh Agarwal, M Jeeva Sankar, Madhan Kumar, Ravi Sharma, Harish Bagh, Sukalyan Das, Kamal Kishore Mundra, Pratima Anand, Akash Sharma, Suksham Jain, Aukifa Islam, Partha Pratim Das, Anuradha Sharma, Vibhor Tak, Kavitha, Amar Singh Thakur, Dhana Ram Gossai, Mangla Sood, Varsha Gupta, Jagdish Chander, Vishnubhatla Sreenivas, Arti Kapil, Preeti Semwal, Vineeta Baloni, Pratibha Singh, Sanjay Kumar, Divashree Jhurani, Varsha Mittal, Girish Mishra, Danish Nafees, Padma Das

**Affiliations:** a All India Institute of Medical Sciences, New Delhi, India; b Jawaharlal Institute of Postgraduate Medical Education & Research, Puducherry, India; c Government Medical College and Hospital, Chandigarh, India; d All India Institute of Medical Sciences, Raipur, India; e Assam Medical College and Hospital, Dibrugarh, India; f All India Institute of Medical Sciences, Jodhpur, India; g Clinical Development Services Agency, Translational Health Science and Technology Institute, Faridabad, India

## Abstract

**Background:**

Neonatal sepsis epidemiology has been adequately reported in tertiary-care hospitals. However, such data are scarce from district hospitals in low-income and middle-income countries. This study aimed to evaluate the incidence of sepsis, pathogen profile, and antimicrobial resistance among neonates admitted to the special newborn care units in district hospitals in India.

**Methods:**

We prospectively enrolled neonates admitted to newborn units in five district hospitals in India between October, 2019, and December, 2021. Blood cultures were obtained from neonates who met prespecified criteria and were processed at the laboratories of the tertiary-care hospitals linked to each district hospital. Identification of pathogens and antimicrobial susceptibility testing was performed using the automated system; all isolates were confirmed using matrix-assisted laser desorption–ionisation-time of flight. The primary outcome was the incidence of culture-positive sepsis. The final label of culture-positive sepsis was assigned based on culture reports and clinical course. Multidrug resistance was defined as resistance to antibiotics in at least three of the six antibiotic classes, including third generation cephalosporins, carbapenems, and aminoglycosides.

**Findings:**

The study enrolled 6612 neonates (3972 inborn [born at the same hospital] and 2640 outborn [referred from other hospitals or homes]). Mean gestation was 37·1 weeks and mean birthweight was 2540 g. 3357 (50·8%) neonates met clinical sepsis criteria. The overall incidence of culture-positive sepsis was 213 (3·2%; 95% CI 0·6–14·4); ranging from 0·6% to 10·0% across the five sites. The incidence was higher in outborn neonates than inborn neonates: 132 [5·0%] versus 81 [2·0%]. The case-fatality rate of culture-positive sepsis was 36·6% (95% CI 12·1–71·0). Gram-negative bacilli accounted for 156 (70·0%) of 223 organisms isolated: *Klebsiella pneumoniae* (51 [22·9%]), *Escherichia coli* (33 [14·8%]), and *Enterobacter* spp (26 [11·7%]) were the most common Gram-negative organisms. 75%–88% of isolates of *K pneumoniae, E coli, Enterobacter* spp, and *Acinetobacter baumannii* were multidrug resistant.

**Interpretation:**

The high incidence of culture-positive sepsis, case-fatality rates, and multidrug resistance among common pathogens underscores an urgent need to strengthen infection prevention and control practices, establish blood culture facilities, and implement antimicrobial stewardship programmes in district-level hospitals in India.

**Funding:**

Bill & Melinda Gates Foundation.

**Translation:**

For the Hindi translation of the abstract see Supplementary Materials section.

## Introduction

Neonatal sepsis results in more than 550 000 deaths globally every year.^1^ India accounts for nearly one-fourth of the global burden of infection-related deaths. Sepsis remains a major hurdle to lowering neonatal mortality rates in low-income and middle-income countries (LMICs).

In a multi-site cohort study done in three tertiary hospitals in Delhi (the Delhi Neonatal Infection Study [DeNIS] collaboration), a high burden of sepsis, sepsis-related mortality, and multidrug resistance among the most commonly reported pathogens were shown to be present.^2^ The study findings helped highlight the need to prevent neonatal sepsis and reduce antimicrobial resistance (AMR) in tertiary hospitals in India. Unfortunately, only a few high-quality multi-site prospective studies have evaluated the epidemiology of sepsis in district hospitals, ie, secondary-care health facilities in LMICs.

More than a million newborns are admitted annually to the 979 special newborn care units (SNCUs) in district hospitals across India.^3^ These SNCUs care for sick newborns, except those requiring invasive mechanical ventilation, inotropes, or surgical interventions. Sepsis is one of the most common causes of admission to SNCUs, which exist only in district hospitals: up to one-fourth of the neonates, particularly those referred from other health facilities or homes, are diagnosed with sepsis.^4^ About 40% of the neonates admitted to the SNCU receive antibiotics during their stay.^5^ However, the SNCUs, save for a few units attached to medical colleges, do not have in-house facilities for performing blood cultures–the gold standard for diagnosing sepsis.^6^ Thus, health-care providers in most SNCUs initiate antibiotics in neonates suspected of sepsis without testing blood cultures. Without information on the local flora and the antibiogram, the choice of antibiotics remains empirical and broad-spectrum, potentially fuelling AMR. Additionally, given the absence of facilities for performing blood cultures in most district hospitals, there are no reliable data on the burden of culture-positive sepsis, pathogen profile, and AMR patterns in SNCUs. The aim of the present study was to fill this gap by examining the clinical and microbiological profile of sepsis in district hospitals.

**Research in context**

**Evidence before this study**

We searched PubMed for articles published from Jan 1, 2006, to Aug 31, 2024, using the search terms “neonate” and “sepsis”. The search was limited to studies from low-income and middle-income countries; no language restrictions were applied. Of the 3866 citations screened, we found 24 studies reporting data on the burden of neonatal sepsis in secondary-care hospitals. We excluded studies reporting data only from tertiary-care hospitals or with more than 50% of neonates from tertiary-care hospitals, those without appropriate denominators (ie, no numbers of admissions or livebirths), and studies focused on single pathogens. 11 studies had no laboratory confirmation of sepsis by culture. Among the remaining 13 studies reporting culture-positive sepsis, eight were from a single centre and had reported routine microbiology and clinical data. Five studies enrolled neonates referred to the study hospitals by community health workers with signs of possible serious bacterial infection. The quality of the included studies was low. The median number of neonates with culture-positive sepsis was 40 (IQR 28–67). In the eight studies reporting livebirths, the incidence of culture-positive sepsis varied from 3 to 45 per 1000 livebirths. Among the six studies reporting the number of admissions, the incidence ranged from 1·1% to 10·5% of all admissions. In two studies, the proportion of early-onset sepsis (ie, onset within 2–6 days after birth) was 54% and 62%. Gram-negative pathogens accounted for 60% (range 30%–83%; nine studies) of isolated organisms. *Klebsiella* spp (seven studies), *Escherichia coli* (five studies), and *Staphylococcus aureus* (seven studies) were the most reported pathogens. Only six studies provided data on antimicrobial resistance patterns. Gram-negative pathogens showed a variable degree of resistance to commonly used antibiotics such as ampicillin (from 70% to 100%; six studies), gentamicin (from 14% to 46%; four studies), and cefotaxime (from 14% to 50%; six studies). Carbapenem resistance was low among *E coli* and *Klebsiella* spp (from 0% to 17%; four studies).

**Added value of this study**

Unlike most studies from single centres that reported routinely collected data, this study is a large, multi-site, prospective observational study conducted in five district hospitals with high methodological rigour, ensuring quality assurance at multiple levels. The study found a high but varying incidence of culture-positive sepsis among the five study sites. Notably, it showed a high case-fatality rate among neonates. The all-cause mortality rate of those with culture-positive sepsis was also higher compared with neonates never suspected to have sepsis or who had culture-negative sepsis. Gram-negative bacilli accounted for 70% of isolated organisms. The proportion of the three most common Gram-negative bacilli—*Klebsiella* spp, *E coli,* and *Enterobacter* spp—that was multidrug resistant is almost comparable to the rates reported from the tertiary hospitals in India.

**Implications of all the available evidence**

Existing literature from small, single-centre studies highlights the need for more information on the burden of culture-positive sepsis and antimicrobial resistance. The high burden of sepsis, case-fatality rates, and multidrug resistance observed in the present study highlights the need to scale up infection prevention and control practices and establish blood culture facilities for reliable diagnosis in district-level hospitals in India.

## Methods

### Study design and setting

In this prospective observational study, we enrolled neonates admitted to five SNCUs of district hospitals located across India: Government Hospital, Cuddalore; District Hospital, Mahasamund; Regional Hospital, Una; Civil Hospital, Sivasagar; and Government Nahata Hospital, Balotra. Each of the SNCUs was linked with a tertiary care institute: the Jawaharlal Institute of Postgraduate Medical Education & Research, Puducherry (with the SNCU in Cuddalore); the All India Institute of Medical Sciences (AIIMS), Raipur (Mahasamund); the Government Medical College & Hospital, Chandigarh (Una); the Assam Medical College, Dibrugarh (Sivasagar); and the AIIMS, Jodhpur (Balotra). The tertiary institutes supervised and mentored the respective district hospital sites, processed the blood cultures, reported antimicrobial sensitivity patterns, and stored and transported the pathogens to the nodal centre. The SNCUs varied from each other in the number of annual admissions, the proportions of outborn neonates admitted, and the staffing and facilities available (appendix 2 p 3).

The study was approved by the Institutional Ethics Committees of AIIMS, New Delhi (IEC-683/07.12.2018, RP-12/2018); the Jawaharlal Institute of Postgraduate Medical Education & Research, Puducherry (JIP/IEC/2019/068); the Government Medical College & Hospital, Chandigarh (GMC/IEC/2019/295); AIIMS, Jodhpur (AIIMS/IEC/2018/1642); Assam Medical College, Dibrugarh (2018/AMC/EC/10); and the State Health Resource Centre, Chhattisgarh (2280/SHRC/2018). The administrative approval for including SNCUs' at the district hospitals was obtained from the respective State Mission Director of the National Health Mission. This study is reported using the STROBE guidelines, and its design was guided by the STROBE-NI framework.^7^

### Study population and methods

All inborn (born at the same hospital) and outborn (referred from other hospitals or homes) neonates admitted to the SNCUs were enrolled after obtaining informed written consent from the parents. Dedicated research staff worked with the clinical team to identify neonates with suspected sepsis based on the presence of prespecified perinatal risk factors or clinical signs such as lethargy, refusal to feed, or severe chest in-drawing (appendix 2 p 4).^8^, ^9^

The research nurses performed the diagnostic tests in all neonates with suspected sepsis before initiating antibiotics. At least a 1 mL blood sample was collected for culture in automated bottles (BD BACTEC [Becton Dickinson, East Rutherford, NJ, USA] or BACT/ALERT, [BioMerieux, Marcy-l'Étoile, France]) and incubated at 37°C before being transported within 24 h of collection to the microbiology laboratory of the mentoring tertiary hospital. More than one culture was collected if the clinical condition deteriorated despite initiating antibiotics or before escalating to antibiotics. Cerebrospinal fluid cultures were sent for a few neonates suspected of sepsis based on the discretion of the clinical team and the feasibility at the study sites. Culture results were communicated to the district hospital investigators within 12–48 h of sample receipt.

The clinical team initiated empiric antibiotic therapy in neonates with suspected sepsis per the SNCU policy (appendix 2 p 3). The duration of antibiotics was decided based on the culture reports and clinical course. Using a pretested case record form, the research nurse prospectively recorded the neonatal characteristics and treatment details until discharge, death, or 28 days of life, whichever was earlier.

### Microbiological processing

Blood culture bottles were incubated in an automated culture system for at least 5 days before being labelled sterile. Samples that were positive for bacterial growth were sub-cultured in 5% sheep blood agar (BioMerieux, Marcy-l'Étoile, France) and McConkey agar (Oxoid, Hampshire, UK). Antimicrobial susceptibility testing was performed per the 2019 Clinical and Laboratory Standards Institute guidelines^10^ using the automated VITEK 2 (BioMerieux, Marcy-l'Étoile, France) system at all hospitals except Government Medical College & Hospital, Chandigarh, which used the Muller Hilton Agar by Kirby disc diffusion method. Antimicrobial susceptibility was reported as susceptible, intermediate, resistant, or not tested for individual antibiotics. Isolates classified as intermediate to an antibiotic were included in the resistant category for analysis, except for colistin: the intermediate sensitivity reported by the automated method for colistin was interpreted as sensitive, given the issues related to minimal inhibitory concentration with the polymyxin disks. Gram-negative pathogens were classified based on their susceptibility to the following antibiotic classes: third generation cephalosporins (any one of cefotaxime, ceftriaxone, or ceftazidime); carbapenems (any of imipenem, meropenem, or ertapenem); aminoglycosides (any of gentamicin, amikacin, or netilmicin); fluoroquinolones (ciprofloxacin); β-lactam–β-lactamase inhibitors (any of amoxicillin–clavulanic acid, piperacillin–tazobactam, or cefoperazone–sulbactam); and polymyxins (colistin). Multidrug resistance was defined as resistance to one or more agents in at least three antibiotic classes.^11^ Confirmatory testing for identification and antimicrobial susceptibility testing patterns of all isolates were performed at AIIMS, New Delhi, using matrix-assisted laser desorption–ionisation-time of flight and VITEK 2, respectively. Discrepant results were re-tested; if still discordant, the results at AIIMS, New Delhi, were considered final.

### Outcome measures

The primary outcome was the incidence of culture-positive sepsis. Secondary outcomes included the incidence of early-onset (within 72 h of birth) and late-onset sepsis (after 72 h of birth), all-cause mortality (death within 28 days of life, regardless of the underlying cause of death), and case-fatality rates (death attributable to sepsis within 28 days of life or 21 days of sepsis suspicion, whichever was earlier), the profile of isolated pathogens, and AMR patterns. We used the National Neonatal Perinatal Database definitions to identify the underlying cause of death among neonates who died before 28 days of life.^12^

Principal investigators at each tertiary hospital and investigators at the respective district hospitals reviewed the records of all neonates with suspected sepsis. They then classified the enrolled neonates into four categories: culture-positive sepsis, culture-negative sepsis, suspected sepsis but not labelled as either culture-positive or culture-negative sepsis, and no suspected sepsis, using the definitions adapted from the National Healthcare Safety Network (appendix 2 pp 4–5).^13^ Neonates with suspected sepsis in whom a known pathogen was isolated in blood or cerebrospinal fluid culture were categorised as having culture-positive sepsis. Neonates were considered to have a second episode of sepsis if they developed symptoms at least 48 h after cessation of antibiotic therapy for the initial episode.

An expert committee at AIIMS (New Delhi) reviewed the records of all neonates with positive cultures to confirm the site investigators' labelling of culture-positive sepsis. The committee members requested additional details on the clinical course of neonates in whom their decision differed from the original labelling. They also discussed the case details with the respective tertiary site and district hospital investigators, if required, and assigned the final label of culture-positive sepsis.

### Quality assurance and monitoring

The Clinical Development Services Agency under the Translational Health Science and Technology Institute of India, Department of Biotechnology, functioned as the monitoring partner and implemented strict quality assurance measures across clinical, microbiology, and data management domains. Those at the Clinical Development Services Agency responsible for monitoring made monthly visits to the SNCUs and microbiology laboratories of the tertiary hospitals to supervise study procedures, monitor adherence to standard operating procedures, and verify recorded data (appendix 2 p 6).

### Statistical analysis

Research nurses recorded data in paper case record forms, which site investigators then cross-checked. Data were entered into an electronic data capture platform, Octalsoft (Glorant, Herndon, VA, USA), and analysed using Stata 15.1 and R (version 4.1.2). Descriptive statistics with frequency estimates were used for baseline characteristics. The primary outcome—incidence of sepsis—was expressed as a proportion with 95% CIs inflated for clustering of neonates within the sites using the svyciprop function from the survey package in R, with the survey design accounting for clustering. Case-fatality rates were calculated by dividing deaths attributed to sepsis by the number of neonates with sepsis. The 95% CI of case-fatality rates was also inflated to adjust for the cluster effect. The number of pathogens isolated from neonates who were culture-positive was expressed as frequencies; if two pathogens were isolated from a single sepsis episode, both were included when counting the number of pathogens.

The Cox proportional hazards model was used as a post-hoc analysis to estimate the hazard ratios (HRs) for all-cause neonatal mortality within 28 days for neonates with culture-positive sepsis, culture-negative sepsis, and suspected sepsis but not labelled as either culture-positive or culture-negative sepsis, compared with neonates without suspected sepsis. The model was adjusted for potential confounders, including birthweight, gestational age, birth asphyxia, and the presence of major congenital malformations. It also accounted for the clustering effect of the sites by incorporating a robust variance estimator using the cluster option from the survival package in R. The Kaplan–Meier survival curves were generated using the survfit function and the ggsurvplot package in R (appendix 2 pp 55–79).

### Role of the funding source

The funder of the study had no role in study design, data collection, data analysis, data interpretation, or writing of the report.

## Results

**Figure 1:**
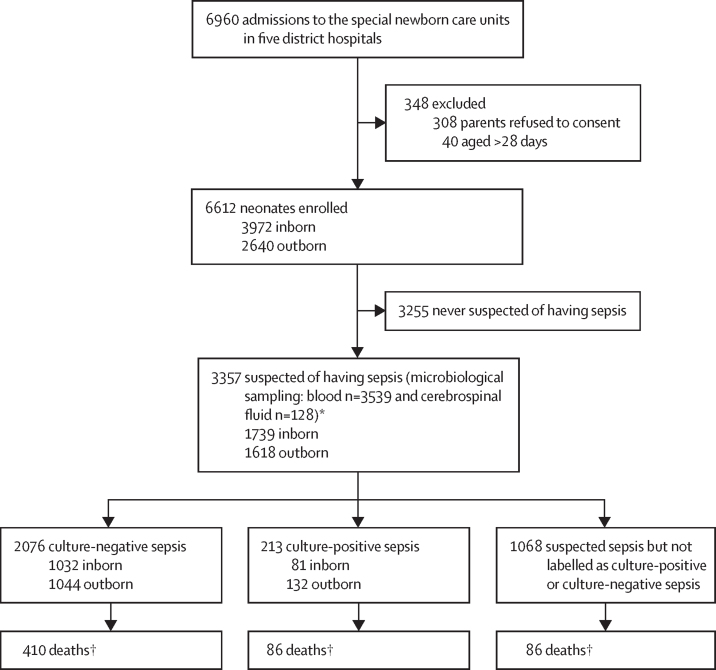
Study flow *More than one sample was obtained during the same episode in a few neonates; one baby in the culture-positive sepsis group had positive cerebrospinal fluid but a negative blood culture report. †Deaths until the 28th day of life.

From October, 2019, to December, 2021 (with intermittent recruitment pauses due to the COVID-19 pandemic), 6960 neonates required admission to the SNCUs, and 6612 (95·0%) were enrolled (figure 1). While 3972 (60·1%) neonates were born in the same hospital (inborn), 2640 (39·9%) were referred from other hospitals or homes (outborn); 175 neonates were born at home (6·6% of outborn neonates; appendix 2 p 7). The mean birthweight across inborn and outborn neonates was 2540 g (SD 690) and mean gestation was 37·1 weeks (SD 2·9; table 1); 1888 (28·6%) of 6612 neonates were born preterm (ie, <37 weeks' gestation). There were substantial inter-site differences in the maternal and neonatal baseline characteristics such as the proportion of caesarean deliveries, preterm births, and outborns admitted (appendix 2 p 8).

**Table 1:** Baseline characteristics

		**Inborn**	**Outborn**
Maternal variables		n=3902	n=2560
	Maternal age, years	25·0 (4·3)	24·9 (4·4)
	Maternal education, years of schooling	11 (8–12)	10 (6–12)
	Monthly family income, Indian rupees	10 000 (6000–12 000)	7000 (5000–10 000)
	Antenatal corticosteroids in <35 weeks of gestation	124/395 (31·4%)	31/510 (6·1%)
	Prolonged rupture of membranes, >18 h	222 (5·7)	267 (10·4)
	Caesarean delivery	1740 (44·6%)	645 (25·2%)
	Maternal antibiotics within 7 days before delivery	1901/3845 (49·4%)	375/2235 (16·8%)
Most common maternal antibiotics received before delivery		n=1901	n=375
	Ampicillin	1037 (54·6%)	10 (2·7%)
	Ceftriaxone or cefotaxime	1294 (68·1%)	94 (25·1%)
Neonatal variables		n=3972	n=2640
	Gestation, weeks*	37·5 (2·6)	36·6 (3·2)
	Preterm birth, <37 weeks gestation	934 (23·5%)	954 (36·1%)
	Birthweight, g†	2620 (654)	2400 (721)
	Small for gestational age	1178/3970 (29·7%)	841/2596 (32·4%)
	Males	2266 (57·0%)	1523 (57·7%)
	Females	1706 (43·0%)	1117 (42·3%)
	Twins or triplets	177 (4·5)	200 (7·6)
	Did not cry at birth	828 (20·8%)	671 (25·4%)
	Meconium-stained liquor	351 (8·8)	294 (11·1)
	Previous hospitalisation (ie, admittance to hospital) other than birth hospitalisation, %	..	133 (5·0)
	Received antibiotics before admission to the study site, %‡	..	134 (5·1)
	Age at admission to special newborn care unit, h	4 (1–72)	12 (3–101)
	Intravenous fluids§	1906 (48·0%)	1525 (57·8%)
	Free flow oxygen§	1539 (38·7%)	1182 (44·8%)
	Continuous positive airway pressure§	271 (6·8)	133 (5·0)
	Mechanical ventilation§	80 (2·0)	71 (2·7)
	Kangaroo mother care*	666 (16·8%)	576 (21·8%)
	Bed-sharing§	532 (13·4%)	681 (25·8%)
	Duration of special newborn care unit stay, days	5 (3–7)	4 (2–7)

Data are n (%), n/N (%), median (IQR), or mean (SD).

* Gestational age of five neonates not known.

† Birthweight of 41 neonates not known.

‡ One outborn neonate received antibiotics on an outpatient basis before admission.

§ Refers to care or intervention anytime during the hospital stay.

Among enrolled neonates, 3357 (50·8%) were suspected of sepsis (figure 1). The most common symptoms prompting suspicion were lethargy and severe chest in-drawing or increased oxygen requirement (appendix 2 p 9). Among all suspected neonates, 213 (6·3%) were labelled to have culture-positive sepsis and 2076 (61·8%) culture-negative sepsis; the remaining neonates (1068; 31·8%) were suspected sepsis but not labelled as either culture-positive or culture-negative sepsis.

The incidence of culture-positive sepsis was 3·2% (213 of 6612; 95% CI 0·6–14·4). It varied among the study sites, ranging from 0·6% to 10·0% (table 2). The incidence was 2·5-fold higher in outborn neonates than inborn neonates: 132 (5·0%) of 2640 versus 81 (2·0%) of 3972 (table 2; appendix 2 p 10). The incidence was inversely proportional to the birthweight and gestation (appendix 2 p 11). 124 (58·2%) of 213 neonates had onset within the first 72 h of life (early onset; appendix 2 p 12). There were 214 episodes of culture-positive sepsis, which amounted to an incidence rate of 5·5 episodes per 1000 patient-days (95% CI 4·8–6·3).

**Table 2:** Incidence and case-fatality rate of culture-positive sepsis

		Incidence*		**Case-fatality rates**†	
		n/N (%)	95% CI	n/N (%)	95% CI
Overall		213/6612 (3·2%)	0·6–14·4‡	78/213 (36·6%)	12·1–71·0‡
	Site 1	10/1742 (0·6%)	0·3–1·0	0/10	0–34·5
	Site 2	135/1358 (9·9%)	8·4–11·7	69/135 (51·1%)	42·4–59·8
	Site 3	22/1020 (2·2%)	1·4–3·2	6/22 (27·3%)	11·6–50·4
	Site 4	34/1100 (3·1%)	2·2–4·3	2/34 (5·9%)	1·0–21·1
	Site 5	12/1392 (0·9%)	0·4–1·5	1/12 (8·3%)	0·4–40·2
Place of birth					
	Inborn	81/3972 (2·0%)	1·6–2·5	21/81 (25·9 %)	17·1–37·1
	Outborn	132/2640 (5·0%)	4·2–5·9	57/132 (43·2%)	34·7–52·1
Onset of sepsis					
	Early onset (within 72 h of birth)	124/6612 (1·9%)	1·6–2·2	51/124 (41·1%)	32·5–50·3
	Late onset (after 72 h of birth)	89/6612 (1·3%)	1·1–1·6	27/89 (30·3%)	21·3–41·1

Case-fatality rate was calculated by dividing the number of deaths due to sepsis within 28 days of life or 21 days of suspected sepsis, whichever is earlier, by the number of neonates in the respective sepsis category; one neonate with one episode each of culture-positive sepsis and culture-negative sepsis was included in the former category.

* Data are number of cases/number of enrolled neonates.

† Data are number of deaths/number of cases (%).

‡ The CI is inflated, accounting for clustering within sites.

**Figure 2:**
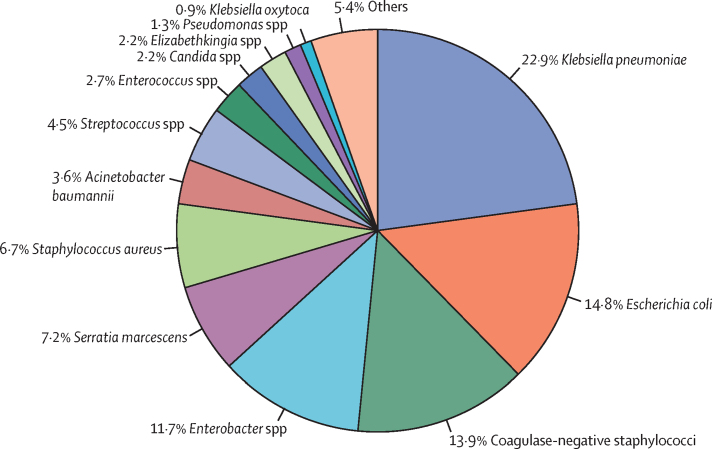
Pathogens isolated from neonates with culture-positive sepsis (n=223) *K pneumoniae* was isolated from the cerebrospinal fluid in one neonate.

Of the 223 pathogens isolated, the most common were *Klebsiella pneumoniae* (51 [22·9%]), *Escherichia coli* (33 [14·8%]), coagulase-negative staphylococci (31 [13·9%]), and *Enterobacter* spp (26 [11·7%])*;* Gram-negative bacilli accounted for 156 (70·0%) of 223 organisms isolated (figure 2; appendix 2 p 13). Group B *Streptococcus* was isolated in two (0·9%) neonates, while *Candida* spp was isolated in five (2·2%). The pathogen profiles were similar between the inborn and the outborn neonates (appendix 2 p 14) and between neonates with early-onset and late-onset sepsis (appendix 2 p 15). However, there was variation in the relative proportion of pathogens isolated on different days of life—for example, *Klebsiella* spp accounted for 20% or less of the total organisms isolated in the first three days of life but 30%–50% on days 4–7; it accounted for less than 5% of the total isolates in the second week of life (appendix 2 p 16). Although the numbers were small, the pathogen profile in neonates born at home was comparable with that of the overall cohort (appendix 2 p 17).

**Figure 3:**
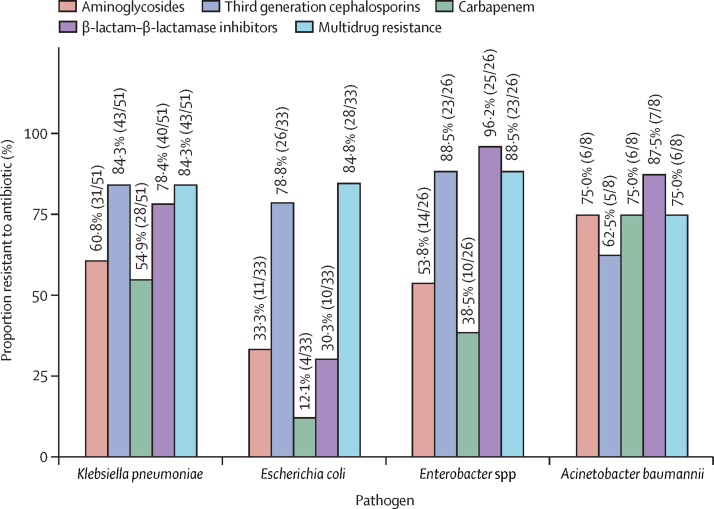
Antimicrobial resistance pattern of common pathogens Multidrug resistance was defined as non-susceptible to one or more agent in any three antimicrobial categories. For the other four categories, resistance was defined as resistance to any antibiotic in the respective class: amikacin, gentamicin, or netilmicin for aminoglycosides; ceftazidime, ceftriaxone, or cefotaxime for third generation cephalosporins; ertapenem, imipenem, or meropenem for carbapenem; and amoxicillin–clavulanic acid, piperacillin–tazobactam, or cefoperazone–sulbactam for β-lactam–β-lactamase inhibitors.

Most pathogens showed resistance to commonly used antibiotics, including β-lactam–β-lactamase inhibitors and carbapenems (figure 3). 43 (84·3%) of 51 isolates of *K pneumoniae*, 28 (84·8%) of 33 isolates of *E coli*, 23 (88·5%) of 26 isolates of *Enterobacter* spp, and six (75·0%) of eight isolates of *Acinetobacter baumannii* were multidrug resistant. Four (7·8%) isolates of *K pneumoniae* were resistant to colistin (appendix 2 p 18). Among Gram-positive pathogens, 17 (60·7%) of 28 isolates of coagulase-negative staphylococci and four (28·6%) of 14 *Staphylococcus aureus* isolates were methicillin-resistant (ie, resistant to cefoxitin; appendix 2 p 19).

The case-fatality rate in neonates with culture-positive sepsis was 36·6% (78 of 213). It varied markedly among the study sites, ranging from 0 to 51·1%, and between inborn and outborn neonates (table 2). The case-fatality rate in neonates with sepsis caused by Gram-negative bacilli was higher than in those caused by Gram-positive cocci (appendix 2 p 20). The case-fatality rate in neonates with sepsis caused by multidrug-resistant or carbapenem-resistant *Klebsiella* spp was 1·5 to 2-fold higher than in neonates with sepsis by isolates sensitive to these antibiotics (appendix 2 p 20). The higher case-fatality rate found in neonates with resistant *Klebsiella* spp isolates was not observed in neonates with resistant *E coli, Enterobacter*, or *Acinetobacter* spp isolates.

2076 (31·4%) neonates were labelled with culture-negative sepsis (appendix 2 p 21). The proportion of neonates with culture-negative sepsis also varied among the individual sites and between inborn and outborn neonates (appendix 2 p 10). The median duration of antibiotics in culture-positive sepsis and culture-negative sepsis was similar (appendix 2 p 22). 88 (4·3%) of 2076 neonates with culture-negative sepsis died secondary to sepsis.

**Figure 4:**
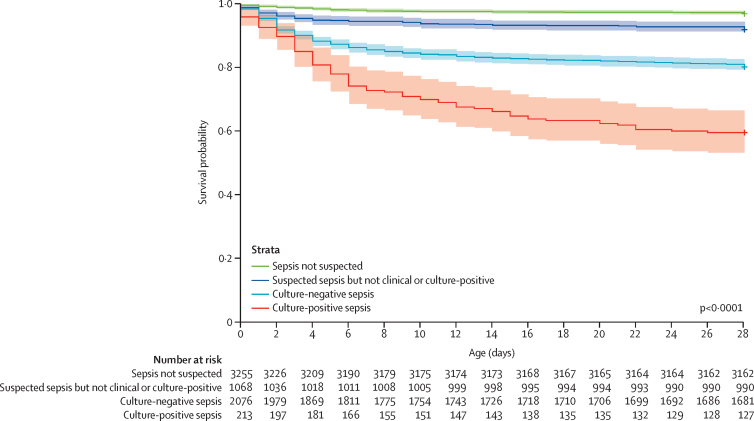
Kaplan–Meier curve for all-cause mortality by sepsis status The shaded areas represent 95% CIs.

Among all enrolled neonates, 676 (10·2%) died during the first 28 days of life (appendix 2 p 23). Sepsis was the underlying cause of death in 179 (27·6%) of the neonates who died (appendix 2 p 24). The all-cause mortality was 40·4% in neonates with culture-positive sepsis and 19·6% in neonates with culture-negative sepsis, compared with 3·0% in neonates never suspected of sepsis (figure 4). The adjusted HRs for all-cause mortality in culture-positive sepsis were 7·4 (95% CI 4·7–11·6) and for culture-negative sepsis 4·6 (2·8–7·5; appendix 2 p 25).

## Discussion

The present study, arguably the largest prospective, multi-site study on neonatal sepsis from district-level hospitals in LMICs, shows four notable findings: a high but varying incidence of culture-positive sepsis at the study sites; a predominance of Gram-negative bacilli among neonates with culture-positive sepsis; a high case-fatality rate, particularly among neonates with sepsis caused by Gram-negative bacilli; and an alarming degree of AMR to even antibiotics such as meropenem, largely unreported from district-level facilities.

The incidence of culture-positive sepsis among inborn neonates was nearly one-third of that reported from tertiary hospitals (2·0% *vs* 6·2%).^2^ Among outborn neonates, the proportion was 5·0%, less than half of that reported from the tertiary hospital of the DeNIS collaboration.^14^ Unlike tertiary hospitals, SNCUs in district hospitals do not cater for neonates who are critically ill, who require invasive ventilation, or who are born extremely preterm. The high burden of sepsis in these facilities, despite caring for relatively stable neonates, is worrisome. Given the increasing number of SNCUs across India and a substantial proportion of admitted neonates being moderate or late preterm who are at a higher risk of sepsis, the high incidence underscores the importance of optimal infection prevention and control practices in these facilities.

The BARNARDS (Burden of Antibiotic Resistance in Neonates from Developing Societies) study conducted in 12 sites across Asia and Africa reported the incidence of laboratory-confirmed sepsis to be 4·69% among all livebirths.^15^ At least six sites were medical colleges or teaching hospitals that likely provided tertiary care for more seriously ill newborns that could explain the higher incidence of culture-positive sepsis than that observed in the present study. The BARNARDS study also reported a marked inter-site variation in the sepsis incidence—from 0·29% to 8·39%.

We observed high case-fatality rates in neonates with culture-positive sepsis among both inborn and outborn neonates (table 2). The high case-fatality rate in the latter group is expected, given the late and selective referral of sick neonates from other health facilities; however, the 25·9% case-fatality rate in inborn neonates is especially concerning. The global NeoOBS (neonatal sepsis observational cohort) study reported an all-cause mortality rate of 17·7% in neonates with culture-positive sepsis but did not provide data on case-fatality rate.^9^ The case-fatality rate in the present study was not uniform across sites—it varied from 0% to 51·1%, reflecting the different baseline characteristics of the enrolled neonates at the sites. For example, the neonates enrolled in site 2 were more premature, had lower birthweights, were more likely to have been referred from another hospital, and were more likely to not have cried at birth compared with other sites (appendix 2 p 8). There were also differences in antenatal corticosteroid exposure, existing infection prevention and control practices, and the availability of skilled health-care professionals.

Previous studies from the district-level facilities in LMICs have reported the predominance of Gram-negative bacilli in neonates with sepsis. A review of neonatal sepsis in south Asia also found *K pneumoniae* and *E coli* to be the most common organisms in these settings.^16^ Gram-negative pathogens were found in 355 (62·9%) of 564 infants with culture-positive sepsis in the NeoOBS study.^9^ The BARNARDS and GERMS-SA studies also showed *Klebsiella* spp as the most frequent pathogen.^15^, ^17^ The predominance of Gram-negative pathogens in south Asian settings has been attributed to suboptimal hygiene and aseptic routines in health facilities.^2^, ^16^, ^18^

Of the 3357 neonates suspected of sepsis in our study, 3042 received at least one dose of parenteral antibiotics. 2076 neonates (31·4% of enrolled neonates) received at least five days of antibiotics and were classified as having culture-negative sepsis. Most of them received a prolonged duration of antibiotics because of the reluctance of health-care providers to stop antibiotics despite a negative blood culture and an improvement in clinical condition. Given the absence of availability of blood culture facilities, the physicians in the district hospitals are not used to relying on the results of microbiological cultures to guide therapeutic decisions. Such prolonged and inappropriate antibiotic use contributes to increasing rates of AMR.

Studies from district hospitals reported high resistance to WHO-recommended first-line antibiotics such as ampicillin, gentamicin, and cefotaxime.^19^, ^20^, ^21^ However, the present study showed up to 70%–80% resistance to even the Watch group of antibiotics, including β-lactam–β-lactamase inhibitors and carbapenems. Up to 80% of the three most common Gram-negative bacilli—*Klebsiella* spp, *E coli,* and *Enterobacter* spp—were multidrug resistant (figure 3). The AMR pattern observed in the district hospitals thus mimics that reported from the tertiary hospitals in India^2^, ^22^ rather than that found in the community settings from Asia.^23^ The latter study–ANISA (Aetiology of Neonatal Infections in South Asia)–showed 83% of the isolates to be susceptible to penicillin, ampicillin, gentamicin, or a combination of these drugs. With the increasing institutional births and overcrowding of facilities often resulting in bed-sharing and a lack of blood culture facilities and protocols for antibiotic initiation and stoppage, the SNCUs risk becoming the hotbeds of infections in India.

The study findings have at least three key policy and research implications. First, there is an urgent need to strengthen infection prevention and control practices in district hospitals, particularly among those with high admission rates, a higher proportion of outborn neonates, and suboptimal nurse–patient ratios. Creating national networks of SNCUs in LMICs for data sharing and benchmarking will help health-care providers learn the best practices of infection prevention and control and other aspects of health care from better-performing units. Also, policy makers must focus on steps to reduce the burden of sepsis and high AMR in neonates in the next National Action Plan for AMR. Second, policy makers must consider establishing blood culture facilities in district hospitals. This is critical given the high and varying incidence of culture-positive sepsis in SNCUs that remain largely undiagnosed because of the absence of blood culture facilities. Third, health-care providers must develop context-specific protocols for identifying and managing sepsis and implement an antimicrobial stewardship model that is feasible, effective, and sustainable in district hospitals. The high burden of culture-negative sepsis underscores the need for better biomarkers or culture-independent methods to identify pathogens and resistance patterns to guide appropriate antibiotic therapy.

The key strength of this study was the establishment of a district hospital–tertiary site dyad, in which a tertiary hospital acted as a mentoring site and provided access to the microbiology laboratory and support to district hospital investigators in study implementation. The study has several limitations. It might have overestimated the incidence of sepsis by coagulase-negative staphylococci, the diagnosis of which was not based on two simultaneous blood cultures. However, the site investigators, followed by a team of experts at the nodal centre, reviewed the history and the clinical course of all neonates with positive blood cultures, including coagulase-negative staphylococci, and assigned the label of culture-positive sepsis. The study sites were from different geographical areas and had varied patient profiles, infrastructures, and practices, which resulted in considerable heterogeneity in the sepsis burden and case-fatality rates; the site with the highest-burden had more outborn neonates referred from catchment areas inhabited by tribal populations. Although the heterogeneity reflects India's diversity, it precludes concluding the epidemiology of sepsis for the whole country.

To conclude, our study findings underscore an urgent need to strengthen infection prevention and control practices, implement context-specific sepsis management protocols and antimicrobial stewardship programmes, and establish blood culture facilities to facilitate reliable diagnosis in district hospitals in India.

### The DH study collaborators

### Contributors

### Data sharing

Data described in the manuscript will be made available upon request to the corresponding author pending approval. Analysis code will be made available upon request with the objective of use.

## Declaration of interests

We declare no competing interests.

## Supplementary Materials

Hindi translation of the abstract
