# Supplementary material for: Multidrug-resistant sepsis in special newborn care units in five district hospitals in India: a prospective cohort study
Source: Lancet Glob Health. 2025 Feb 26;13(5):e870–8. doi: 10.1016/S2214-109X(24)00564-3 (PMC12021664; doi:10.1016/S2214-109X(24)00564-3)
Supplement: Hindi translation of the abstract [file mmc1.pdf]

THE LANCET
Global Health

Supplementary appendix 1

This translation in Hindi was submitted by the authors and we reproduce it as supplied. It has not been peer reviewed. *The Lancet*'s editorial processes have only been applied to the original in English, which should serve as reference for this manuscript.

हिंदी में यह अनुवाद लेखकों द्वारा प्रस्तुत किया गया था और हम इसे जैसे उपलब्ध कराया गया वैसे पुन: पेश करते हैं। इस पर सहकर्मी की समीक्षा नहीं की गई है। लैंसेट की संपादकीय प्रक्रियाओं को केवल अंग्रेजी में मूल पर लागू किया गया है, जो इस पांडुलिपि के संदर्भ के रूप में काम आना चाहिए।

Supplement to: Jain K, Kumar V, Plakkal N, et al. Multidrug-resistant sepsis in special newborn care units in five district hospitals in India: a prospective cohort study. *Lancet Glob Health* 2025; published online Feb 26. https://doi.org/10.1016/S2214-109X(24)00564-3.

सारांश

पृष्ठभूमि:

निम्न और मध्यम आय वाले देशों जैसे की भारत के नवजात शिशुओं में सेप्सिस (इंफेक्शन) पर बड़े अस्पतालों (मेडिकल कॉलेज/ लेवल-III अस्पताल) में काफी शोध किया गया है, लेकिन छोटे अस्पतालों (जिला अस्पताल/ लेवल-II यूनिट) में इस जानकारी का अभाव है। इस अध्ययन का उद्देश्य भारत के जिला अस्पतालों में भर्ती नवजात शिशुओं में सेप्सिस की दर, संक्रमण के कीटाणुओं और उनके एंटीमाइक्रोबियल रेजिस्टेंस (AMR) की स्थिति को समझना था।

तरीका:

अक्टूबर 2019 से दिसंबर 2021 के बीच, पांच जिला अस्पतालों में भर्ती शिशुओं को इस अध्ययन में शामिल किया गया। जिन शिशुओं में सेप्सिस के लक्षण थे, उनसे खून के नमूने लिए गए और उन्हें संबंधित बड़े अस्पतालों की प्रयोगशालाओं में जांच के लिए भेजा गया। संक्रमण के जीवाणुओं की पहचान और उनके एंटीमाइक्रोबियल रेजिस्टेंस की जांच मशीन से की गई। सभी नमूनों की पुष्टि MALDI-TOF तकनीक से हुई। अगर कोई बैक्टीरिया छह में से कम से कम तीन तरह की मुख्य एंटीबायोटिक दवाओं (जैसे 3rd जनरेशन सेफालोस्पोरिन, कार्बापेनेम, और एमिनोग्लाइकोसाइड) से रेसिस्टेंट पाया गया, तो उसे मल्टीड्रग-रेसिस्टेंट (MDR) माना गया।

परिणाम:

इस अध्ययन में 6,612 नवजात शिशुओं (3,972 अस्पताल में जन्मे [इन्बॉर्न] और 2,640 बाहर जन्मे [ऑउटबॉर्न]) को शामिल किया गया। औसत गर्भावधि 37.1 सप्ताह और जन्म के समय वजन 2,540 ग्राम था।

आधे (50.8%) नवजात शिशुओं में सेप्सिस के लक्षण पाए गए। खून में संक्रमण (कल्चर पॉजिटिव सेप्सिस) की दर 3.2% थी, जोकि पांचो अस्पतालों में अलग-अलग (0.6% से 10%) थी। ऑउटबॉर्न शिशुओं में सेप्सिस के मामले (5.0%) इन्बॉर्न शिशुओं (2.0%) से ज्यादा थे। कल्चर पॉजिटिव सेप्सिस वाले शिशुओं की मृत्यु दर 36.6% थी। संक्रमण फैलाने वाले जीवाणुओं में तीन मुख्य बैक्टीरिया- क्लेबसिएल्ला निमोनिए (51 [22.9%]), ई. कोलाई (33[14.8%]), और एन्टेरोबेक्टर प्रजातियाँ (26[11.7%]) थे। इन बैक्टीरिया में ज्यादातर (75%-88%) मल्टीड्रग-रेसिस्टेंस थे।

निष्कर्ष:

भारत के जिला अस्पतालों नवजात शिशुओं में सेप्सिस की ऊँची दर एवं प्रभावित शिशुओं में उच्च मृत्यु दर पाई गई है। संक्रमण फैलाने वाले बैक्टीरिया में मल्टीड्रग-रेसिस्टेंस की भी ऊँची दर पाई गई। इसकी गंभीरता को देखते हुए, जिला अस्पतालों में संक्रमण रोकने के उपाय, और एंटीबायोटिक के सही उपयोग करने की तत्काल आवश्यकता है।
