# Supplementary material for: Multidrug-resistant sepsis in special newborn care units in five district hospitals in India: a prospective cohort study
Source: Lancet Glob Health. 2025 Feb 26;13(5):e870–8. doi: 10.1016/S2214-109X(24)00564-3 (PMC12021664; doi:10.1016/S2214-109X(24)00564-3)
Supplement: Supplementary appendix 2 [file mmc2.pdf]

THE LANCET
Global Health

Supplementary appendix 2

This appendix formed part of the original submission and has been peer reviewed. We post it as supplied by the authors.

Supplement to: Jain K, Kumar V, Plakkal N, et al. Multidrug-resistant sepsis in special newborn care units in five district hospitals in India: a prospective cohort study. *Lancet Glob Health* 2025; published online Feb 26. https://doi.org/10.1016/S2214-109X(24)00564-3.

Burden of multidrug-resistant neonatal sepsis in level-II newborn units of district hospitals in India

Supplementary Annexures

Table of contents

**Annexure 1**		Page no
Supplementary table 1	Details of study sites	3
Supplementary box 1	Definitions used in the study	4
Supplementary figure 1	Neonatal sepsis classification used in the study	5
Supplementary figure 2	Quality assurance	6
Supplementary table 2	Previous hospitalization and antibiotic history for outborn neonates	7
Supplementary table 3	Site-wise baseline characteristics	8
Supplementary table 4	Clinical features at suspicion among neonates with sepsis	9
Supplementary table 5	Burden of culture-positive sepsis by place of birth	10
Supplementary table 6	Burden of culture-positive sepsis by gestation and birth weight	11
Supplementary table 7	Burden of sepsis by onset of sepsis	12
Supplementary table 8	Overall and site-wise pathogen profile	13
Supplementary table 9	Pathogen profile among inborn vs. outborn neonates	14
Supplementary table 10	Pathogen profile based on onset of sepsis	15
Supplementary figure 3	Pathogen profile based on the day of isolation among neonates with culture-positive sepsis (n=223)<br>3A: Pathogen profile among all enrolled neonates<br>3B: Pathogen profile among inborn neonates<br>3C: Pathogen profile among outborn neonates	16
Supplementary table 11	Pathogen profile among home deliveries	17
Supplementary table 12	Antimicrobial resistance pattern of common Gram-negative pathogens	18
Supplementary table 13	Antimicrobial resistance pattern of common Gram-positive pathogens	19
Supplementary table 14	Antimicrobial resistance (AMR) and case-fatality rates by AMR pattern	20
Supplementary table 15	Incidence and case-fatality rate (CFR) of culture-negative sepsis	21
Supplementary table 16	Sepsis categories and duration of antibiotic therapy	22
Supplementary table 17	Neonatal mortality (death by 28 days of age) by place of birth: site-wise	23
Supplementary table 18	Single most underlying cause of death (until 28 days of life)	24
Supplementary table 19	Sepsis categories and the risk of mortality	25
**Annexure 2**: Standard Operating Procedures		26 - 54
**Annexure 3**: Statistical Analysis Plan		55 - 79

Supplementary table 1: Details of study sites

Site	Number of beds	Annual admissions (baseline; before the start of the study)	Staffing	Interventions available	Infection control practices	SNCU empirical antimicrobial policy during the study period
Site 1	20	Live births: 5860<br>Admissions: 996<br>[**2018**]	8 Pediatricians (on rotation)<br>13 Nurses<br>6 Workers/security<br>1 DEO	a) CRP testing<br>b) Hemogram<br>c) CPAP<br>d) Ventilator<br>e) Microbiology lab	Guidelines for preventing infections: **Yes**<br>Antimicrobial policy in place: **Yes**<br>Regular HAI surveillance is conducted: **Yes**	First line: Ampicillin + gentamicin (intramural infants) or cefotaxime + amikacin (extramural infants)<br>2. Second line: Piperacilllin-tazobactam + amikacin<br>3. Third line: Vancomycin + meropenem
Site 2	12	Live births: 1821<br>Admissions: 1100<br>[**2018**]	1 Doctor<br>12 Nurses<br>3 Attendants	Facility for CRP (Qualitative) and hemogram testing	Guidelines for preventing infections: **Yes**<br>Antimicrobial policy in place: **Yes**<br>Regular HAI surveillance is conducted: **No**	First line: Ampicillin + gentamicin<br>Second line: Piperacillin-tazobactam;
Site 3	12	Admissions: Around 720	2 Doctors<br>7 Nurses<br>1 *Yashodha**<br>(traditional birth attendant)<br>2 Cleaning staff<br>1 Watchman	Facility for CRP and hemogram testing	Guidelines for preventing infections: **Yes**<br>Antimicrobial policy in place: **No**#<br>Regular HAI surveillance is conducted: **No**	First line: Ampicillin/cefotaxime + amikacin (cefotaxime was used in outborns)<br>Second line: Piperacillin-tazobactam was added to the ongoing therapy without stopping any of the previous antibiotics<br>Third line: Vancomycin + piperacillin-tazobactam + amikacin
Site 4	12	Admissions: 1035 (**2016**)	3 Pediatricians<br>11 Nurses	Facility for CRP and hemogram testing	Guidelines for preventing infections: **Yes**<br>Antimicrobial policy in place: **Yes**<br>Regular HAI surveillance is conducted: **Yes**	First line: Piperacillin-tazobactam<br>Second line: Vancomycin + piperacillin-tazobactam
Site 5	8	Live births: 3919<br>Admissions: 755<br>[**2018**]	3 Pediatricians<br>8 Nurses	Facility for CRP and hemogram testing	Guidelines for preventing infections: **Yes**<br>Antimicrobial policy in place: **No**#<br>Regular HAI surveillance is conducted: **No**	First line: Cefotaxime and amikacin<br>Second line: Meropenem

CPAP= Continuous positive airway pressure; CRP= C-reactive protein; DEO= Data entry operator; HAI= healthcare-associated infections

**Yashodhas are semi-skilled workers who provide support and care to mothers and newborns during their stay at the district hospital; they assist the nurse in providing various non-clinical activities*

#Sites 3 and 5 did not have a written antimicrobial policy.

Supplementary box 1: Definitions used in the study

**Suspected sepsis**	Age of the neonate <28 days<br>AND<br>Presence of any one of the risk factors/ clinical symptoms/signs from the following list, for which the neonate has no other known or reliable explanation (to be verified independently by a neonatologist from the corresponding tertiary site later):<br>***Perinatal risk factors***<br>1. Foul-smelling liquor<br>2. At least two of the following: (i) spontaneous prematurity and preterm pre-labor rupture of membranes (PPROM; irrespective of duration of rupture of membranes) (ii) Febrile illness in the mother with suspected bacterial infection warranting start of antibiotics by attending clinician in the week prior to delivery (iii) rupture of membranes >24 hours (iv) unclean vaginal examination(s) during labor<br>***Clinical symptoms/signs***<br>3. No movement or movement only when stimulated<br>4. Refusal to feed<br>5. Severe chest in-drawing or increased oxygen requirement or need for respiratory support<br>6. Grunting<br>7. New onset apnea or increased severity or frequency of apnea in a baby who already is having apneas<br>8. Cyanosis or desaturation needing oxygen therapy or respiratory support (or increase in oxygen requirement/deterioration in a baby who is already receiving oxygen therapy)<br>9. Fever or hypothermia (>37·5˚C or <36·5˚C)<br>10. Tachycardia or episodes of bradycardia (>180/min or <100/min)<br>11. Capillary refill time (CRT) > 3 sec<br>12. Mottled skin or other evidence of shock<br>13. Erythema in the skin around umbilical stump (extending to >1 cm)<br>14. Lethargy or drowsiness<br>15. Convulsions<br>16. Abnormal posturing<br>17. Hypotonia or floppiness<br>18. Bulging fontanelle<br>19. Vomiting or abdominal distension<br>20. Bleeding<br>21. Sclerema
**Culture-positive sepsis**	Neonate with 'suspected sepsis' (see above) AND isolation of a recognized pathogen* from blood, cerebrospinal fluid, or other body fluids.<br>If *CONS**, it should be cultured from one or more blood samples drawn on separate occasions and the organism cultured from blood is not related to an infection at another site.
**Culture-negative sepsis**	Neonate with 'suspected sepsis' AND negative blood culture or blood culture deemed to have grown a commensal AND clinical course consistent with sepsis or the neonate received at least 5 days of antibiotics
**Suspected but not labelled as either culture-positive or culture-negative sepsis**	Neonate with 'suspected sepsis' AND negative blood culture AND (clinical course not consistent with sepsis OR any other attributable cause identified for the signs) AND the neonate received antibiotics for less than 5 days
**Early-onset sepsis**	Occurrence (time of onset of symptoms) of culture-positive or culture-negative sepsis at or before 72 h of life
**Late-onset sepsis**	Occurrence of culture-positive or culture-negative sepsis after 72 h of life
**Meningitis**	Positive cerebrospinal fluid culture, Gram staining, or neutrophilic leukocytosis, with or without low glucose (<50% of plasma glucose level) and high protein content<br>(Normal range of CSF components: cells - up to 30 cells/mm^3; polymorphonuclear leukocytes (PMN) - 60%; protein: up to 150 mg/dL; CSF/blood glucose >60%
**Systemic fungal infection**	Blood culture, CSF, or suprapubic urine positive for yeasts or presence of budding yeast/hyphae AND physician institutes appropriate therapy for fungal infection
**New episode of sepsis**	When the neonate becomes symptomatic after 48 hours of stopping appropriate antibiotic therapy.
**Case-fatality rate**	Death due to sepsis within 28 days of life or 21 days of suspicion, whichever is earlier. For each death, the single-most underlying cause of death was assigned by the investigator based on the criteria outlined in National Neonatal Perinatal database (NNPD)

Supplementary figure 1: Neonatal sepsis classification used in the study

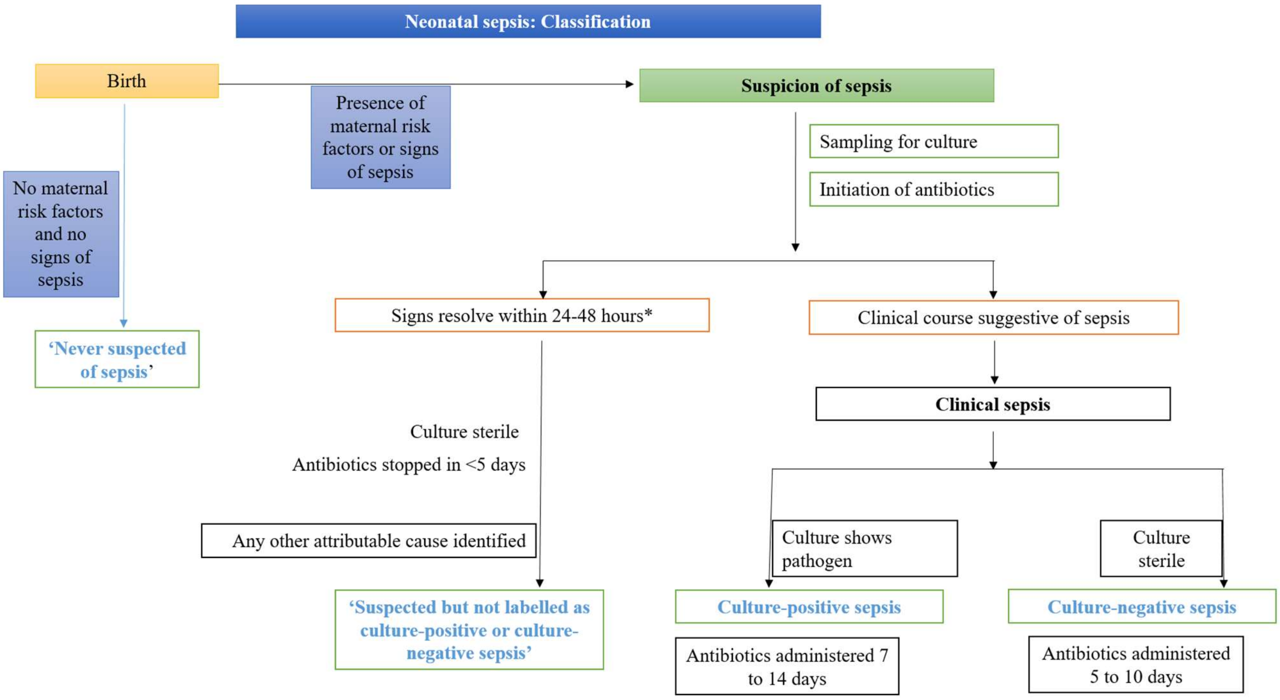

*If the signs resolved within 48 hours, and culture was positive, it was still labelled as 'suspected but not labelled as culture-positive or culture-negative sepsis'.

Supplementary figure 2: Quality assurance

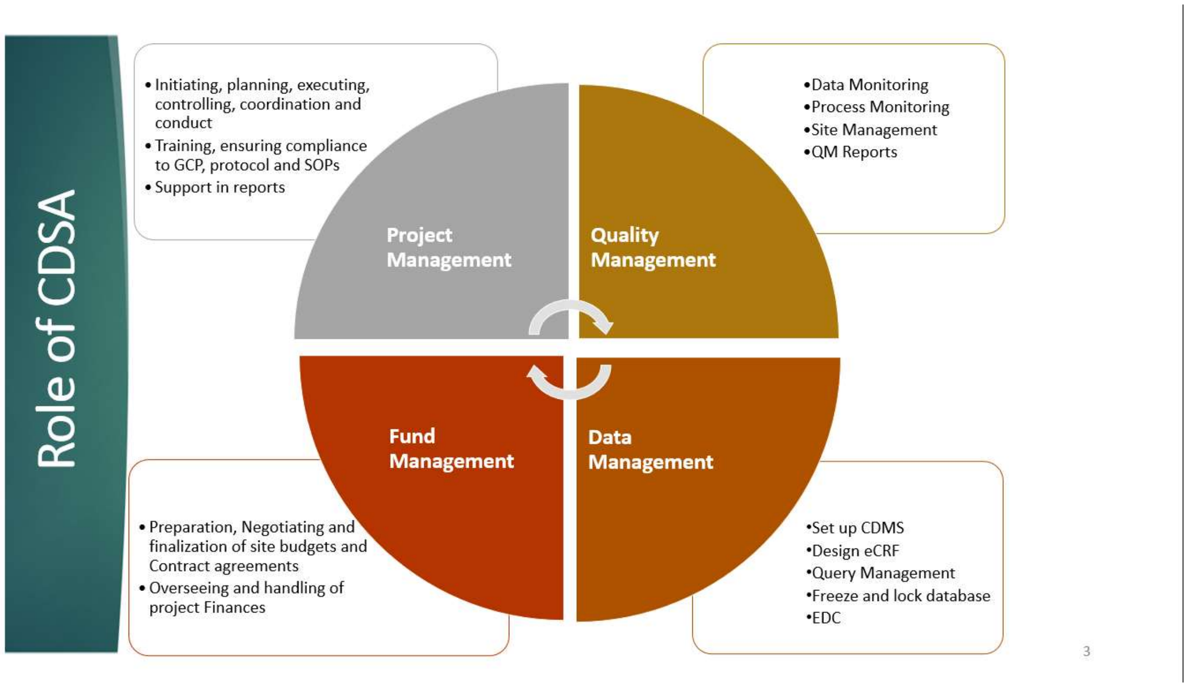

The quality management team maintained high standards of data reliability and integrity, with over 5,84,251 variables monitored and more than 33,309 observations reviewed. The central and on-site monitoring efforts raised 25,063 and 8,157 queries respectively, with most involving data entry discrepancies and less than 0.3% categorized as protocol deviations

Supplementary table 2: Previous hospitalization and antibiotic history for outborn neonates

Variable	n = 2640
**Place of delivery**	
• Home	175 (6·6)
• Hospital	2419 (91·6)
• Others	46 (1·8)
**Delivery attended by:**	
• Doctor/Nurse/MW	2440 (92·5)
• Traditional birth attendant	54 (2·0)
• Family member	93 (3·5)
• Others	53 (2·0)
Previous hospitalization other than birth hospitalization (%)	133 (5·0)
Duration of previous hospitalization (hrs)	48 (20-120)
**Type of hospital where the baby was admitted***	
• Primary health facility	39 (28·9)
• Large private hospital	32 (23·7)
• Small private NH	22 (16·3)
• District hospital	21 (15·6)
• Tertiary hospital	20 (14·8)
• Others	1 (0·7)
Received antibiotics** (%)	134 (5·1)
Duration of antibiotics received in previous hospitalization *(days)*	3 (2-8)

Data are n (%), median (IQR), mean (SD), or n/N (%).
**Two neonates had two previous hospital admissions.*
***One neonate received antibiotics on an outpatient basis before admission.*

Supplementary table 3: Site-wise baseline characteristics

Characteristics	Site 1	Site 2	Site 3	Site 4	Site 5
Maternal variables	***n=1715***	***n=1300***	***n=988***	***n=1092***	***n=1367***
Maternal age (years)	24·8 (4·1)	24·1 (3·9)	24·2 (4·1)	24·2 (4·5)	27·2 (4·3)
Maternal education (years of schooling)	12 (10-15)	9 (7-12)	5 (0-9)	10 (7-12)	12 (10-15)
Median monthly family income (INR)	10000 (7000-15000)	5000 (4000- 6000)	10000 (7000-10000)	8000 (6000- 11000)	10000 (8000- 20000)
**Antenatal corticosteroids (in <35 weeks of gestation)**<br>• **Any**<br>• **None**	**n=100/111**<br>**63 (63)**<br>**37 (37)**	**n= 386/388**<br>**10 (2·6)**<br>**376 (97·4)**	**n=192/201**<br>**46 (24·0)**<br>**146 (76·0)**	**n=116/117**<br>**5 (4·3)**<br>**111 (95·7)**	**n=111/113**<br>**31 (27·9)**<br>**80 (72·1)**
**Prolonged rupture of membranes (>18 h)**	**28 (1·6)**	**271 (20·8)**	**22 (2·2)**	**9 (0·8)**	**159 (11·6)**
**Caesarean delivery**	**934 (54·5)**	**241 (18·5)**	**96 (9·7)**	**328 (30·0)**	**786 (57·5)**
**Maternal antibiotics within 7 days before delivery**<br>***Commonest antibiotics***<br>• **Ampicillin**<br>• **Ceftriaxone/cefotaxime**	**1247 (72·7)**<br><br>**1041 (83·5)**<br>**653 (52·4)**	**59 (4·5)**<br><br>**5 (8·5)**<br>**48 (81·4)**	**11 (1·1)**<br><br>**0**<br>**6 (54·5)**	**154 (14·1)**<br><br>**1 (0·7)**<br>**140 (90·9)**	**805 (58·9)**<br><br>**0**<br>**541 (67·2)**
Neonatal variables	***n=1742***	***n=1358***	***n=1020***	***n=1100***	***n=1392***
Gestation (weeks)	37·7 (2·1)	36·2 (4·1)	36·1 (2·6)	38·2 (2·7)	37·2 (2·2)
**Preterm birth (<37 weeks' gestation)**	**340 (19.5)**	**587 (43.2)**	**376 (36.9)**	**242 (22.0)**	**343 (24.6)**
**Birth weight (g)**	**2680 (622)**	**2160 (734)**	**2420 (684)**	**2650 (632)**	**2730 (610)**
Small-for-gestational age	544 (31·2)	564 (41·5)	235 (23·0)	408 (37·1)	268 (19·3)
Boys	975 (56·0)	765 (56·3)	621 (60·9)	632 (57·5)	796 (57·2)
**Outborn**	**321 (18·4)**	**1044 (76·9)**	**560 (54·9)**	**197 (17·9)**	**518 (37·2)**
Twins/Triplets	63 (3·6)	144 (10·6)	87 (8·5)	23 (2·1)	60 (4·3)
**Did not cry at birth**	**235 (13·5)**	**646 (47·6)**	**295 (28·9)**	**211 (19·2)**	**112 (8·0)**
Meconium-stained liquor	173 (9·9)	237 (17·4)	67 (6·6)	72 (6·5)	96 (6·9)
Previous hospitalization other than birth hospitalization (%)	24 (1·4)	24 (1·8)	27 (2·6)	22 (2·0)	36 (2·6)
Received antibiotics before admission at study site (%)	37 (2·1)	38 (2·8)	28 (2·7)	27 (2·5)	4 (0·3)
Age at admission in SNCU (hrs)	4·3 (0·7-68·3)	3·2 (1·2-14·8)	4·8 (1·2-68·7)	30·4 (1·1-71·9)	75·7 (2·7-118)
Care-related variables					
IV fluids[*]	920 (52·8)	985 (72·5)	817 (80·1)	376 (34·2)	333 (23·9)
Free flow oxygen[*]	756 (43·4)	864 (63·6)	552 (54·1)	254 (23·1)	295 (21·2)
Continuous positive airway pressure (CPAP)[*]	161 (9·2)	62 (4·6)	66 (6·5)	10 (0·9)	105 (7·5)
Mechanical ventilation[*]	63 (3·6)	46 (3·4)	40 (3·9)	0	2 (0·1)
Readmissions[†]	21/1660 (1·3)	23/1146 (2·0)	13/928 (1·4)	13/1079 (1·2)	85/1350 (6·3)
Pre-lacteal feeds	12 (0·7)	1 (0·1)	3 (0·3)	1 (0·1)	158 (11·4)
Kangaroo mother care (KMC)[*]	403 (23·1)	389 (28·6)	311 (30·5)	69 (6·3)	70 (5·0)
Bed-sharing[*]	7 (0·4)	533 (39·2)	415 (40·7)	176 (16·0)	82 (5·9)
Duration of SNCU stay (days)	6 (4- 8)	4 (2- 8)	5 (4- 8)	4 (3- 6)	4 (3- 6)

Data are n (%), median (IQR), mean (SD), or n/N (%).
INR=Indian rupees. SNCU=special newborn care unit.
[*]Received the care/intervention anytime during the hospital stay.
[†]Deaths during first hospitalization and still-admitted neonates at the end of the study were removed from the denominator.
p-value was < 0.001 for all the maternal and neonatal variables except 'gender'.

Supplementary table 4. Clinical features at suspicion among neonates with sepsis

	Clinical features at suspicion	Suspected of sepsis n=3357	Culture-positive sepsis n=213	Culture-negative sepsis n=2076
1	Lethargy or drowsiness	2248 (67.0)	168 (78.9)	1499 (72.2)
2	Severe chest wall in-drawing, increased oxygen requirement or need for ventilation	2062 (61.4)	140 (65.7)	1286 (61.9)
3	Difficulty feeding or feeding intolerance	938 (27.9)	54 (25.4)	578 (27.8)
4	Grunting	909 (27.1)	44 (20.7)	533 (25.7)
5	Convulsions	581 (17.3)	30 (14.1)	384 (18.5)
6	Abnormal temperature (>37.5C or < 36.5C) or temperature instability (e.g., wide variations)	554 (16.5)	29 (13.6)	383 (18.4)
7	No movement or movement only when stimulated	303 (9.0)	27 (12.7)	175 (8.4)
8	Cyanosis	247 (7.4)	17 (8.0)	157 (7.6)
9	Apnea	206 (6.1)	35 (16.4)	125 (6.0)
10	Abdominal distension/vomiting	225 (6.7)	25 (11.7)	132 (6.4)
11	Hypotonia/floppiness	165 (4.9)	13 (6.1)	96 (4.6)
12	Abnormal heart rate (>180 beats/min or < 100 beats/min)	135 (4.0)	20 (9.4)	78 (3.8)
13	Capillary refill time >3 sec or mottled skin or other evidence of shock	131 (3.9)	14 (6.6)	56 (2.7)
14	Bleeding from any site	54 (1.6)	10 (4.7)	30 (1.4)
15	Abnormal posturing	15 (0.4)	1 (0.5)	12 (0.6)
16	Sclerema	20 (0.6)	5 (2.3)	10 (0.5)
17	Bulging fontanelle	11 (0.3)	1 (0.5)	7 (0.3)

Data are n (%).

Supplementary table 5: Burden of sepsis by place of birth

	Inborn		Outborn	
	**N**	**n (%; 95% CI)**	**N**	**n (%; 95% CI)**
**Culture-positive sepsis**	**3972**	**81 (2·0; 0·05-7·0)***	**2640**	**132 (5·0; 1·1-19·0)***
• Site 1	1421	7 (0·5; 0·2-1·0)	321	3 (0·9; 0·2-2·7)
• Site 2	314	30 (9·6; 6·5-13·4)	1044	105 (10·1; 8·3-12·0)
• Site 3	460	10 (2·2; 1·1-4·0)	560	12 (2·1; 1·1-3·7)
• Site 4	903	26 (2·9; 1·9-4·2)	197	8 (4·1; 1·8-7·9)
• Site 5	874	8 (0.9; 0·4-1.8)	518	4 (0·8; 0·2-2·0)
**Culture-negative sepsis**	**3972**	**1032 (26·0; 21·3-31·0)***	**2640**	**1044 (39·5; 25·1-56·0)***
• Site 1	1421	349 (24·6; 22·3-26·9)	321	105 (32·7; 27·6-38·1)
• Site 2	314	109 (34·7; 29·5-40·3)	1044	500 (47·9; 44·8-51·0)
• Site 3	460	154 (33·5; 29·2-38·0)	560	253 (45·2; 41·0-49·4)
• Site 4	903	234 (25·9; 23·1-28·9)	197	77 (39·1; 32·2-46·3)
• Site 5	874	186 (21·3; 18·6-24·1)	518	109 (21·0; 17·6-24·8)

Data are total number of neonates and number of cases (%; 95% CI).

**The confidence intervals are inflated, accounting for clustering within sites.*

Supplementary table 6: Burden of culture-positive sepsis by gestation and birth weight

	Inborn	Outborn*	Total*
Overall	81/3972 (2·0)	132/2640 (5·0)	213/6612 (3·2)
<28 week	3/28 (10·7)	0/20 (0)	3/48 (6·3)
28-31 week	12/110 (10·9)	24/203 (12·3)	36/313 (11·8)
32-33 week	6/149 (4·0)	18/219 (8·2)	24/368 (6·5)
34-36 week	12/647 (1·9)	20/512 (3·9)	32/1159 (2·8)
37 weeks & above	48/3036 (1·6)	70/1683 (4·2)	118/4719 (2·5)
<1000 g	7/37 (18·9)	4/56 (7·1)	11/93 (11·8)
1000-1499 g	11/157 (7·0)	23/270 (8·6)	34/427 (8·0)
1500-2499 g	26/1263 (2·1)	48/882 (5·6)	74/2145 (3·5)
2500 g & above	37/2515 (1·5)	53/1391 (3·8)	90/3906 (2·3)

Data are n/N (%).

**The gestational age of 5 neonates in the total cohort (2 and 3 neonates in the inborn and outborn cohorts, respectively) was not known; the birth weight of 41 neonates in the total cohort (all from the outborn cohort) was not available.*

Supplementary table 7: Burden of sepsis by onset of sepsis

	**Early-onset sepsis**	**Late-onset sepsis**
	**Culture-positive sepsis (n= 213)**	
**Overall (n=213)***	**124 (58.2; 50·6-65·0)**	**89 (41.8; 34·6-49·0)**
Site 1 (n=10)	6 (60.0; 27·4-86·3)	4 (40.0; 13·7-72·6)
Site 2 (n=135)	81 (60.0; 51·2-68·2)	54 (40.0; 31·8-48·8)
Site 3 (n=22)	9 (40.9; 21·5-63·3)	13 (59.1; 36·7-78·5)
Site 4 (n=34)	22 (64.7; 46·5-80·0)	12 (35.3; 20·3-53·5)
Site 5 (n=12)	6 (50.0; 25·4-74·6)	6 (50.0; 25·4-74·6)
	**Culture-negative sepsis (n= 2076)**	
**Overall (n=2076)***	**1638 (78.9; 64·6-88·0)**	**438 (21.1; 11·6-35·0)**
Site 1 (n=454)	357 (78.6; 74·5-82·2)	97 (21.4; 17·7-25·5)
Site 2 (n=609)	539 (88.5; 85·6-90·9)	70 (11.5; 9·1-14·4)
Site 3 (n=407)	281 (69.0; 64·3-73·5)	126 (31.0; 26·5-35·7)
Site 4 (n=311)	261 (83.9; 79·3-87·7)	50 (16.1; 12·3-20·2)
Site 5 (n=295)	200 (67.8; 62·1-73·0)	95 (32.2; 27·0-37·9)

Data are n (%), or n/N (%).
Early-onset and late-onset sepsis were defined as those occurring at or before 72 hours and after 72 hours of birth, respectively.
**The confidence intervals are inflated, accounting for clustering within sites.*

Supplementary table 8: Overall and site-wise pathogen profile

Pathogens	Site 1 (n=12)	Site 2 (n=141)	Site 3 (n=22)	Site 4 (n=35)	Site 5 (n=13)	Overall (n=223)
**Gram-negative bacteria**						
Klebsiella pneumoniae	2 (16·7)	40 (28·4)	4 (18·2)	5 (14·3)	0	51 (22·9)
Klebsiella oxytoca	0	2 (1·4)	0	0	0	2 (0·9)
Escherichia coli	1 (8·3)	23 (16·3)	6 (27·3)	1 (2·8)	2 (15.4)	33 (14·8)
Enterobacter cloacae	1 (8·3)	11 (7·7)	0	2 (5·7)	0	14 (6·3)
Enterobacter hormaechei	0	1 (0·7)	1 (4·5)	5 (14·3)	0	7 (3·1)
Enterobacter asburiae	0	5 (3·5)	0	0	0	5 (2·2)
Serratia marcescens	0	15 (10·6)	1 (4·5)	0	0	16 (7·2)
Acinetobacter baumannii	0	3 (2·1)	3 (13·6)	2 (5·7)	0	8 (3·6)
Acinetobacter radioresistens	0	0	0	1 (2·8)	0	1 (0·4)
Elizabethkingia anophelis	0	3 (2·1)	0	0	0	3 (1·3)
Elizabethkingia meningoseptica	0	2 (1·4)	0	0	0	2 (0·9)
Pseudomonas aeruginosa	0	1 (0·7)	1 (4·5)	0	0	2 (0·9)
Pseudomonas stutzen	0	0	0	1 (2·8)	0	1 (0·4)
Achromobacter xylosoxidans	0	0	0	0	1 (7·7)	1 (0·4)
Aeromonas caviae	0	1 (0·7)	0	0	0	1 (0·4)
Burkholderia cepacia	0	1 (0·7)	0	0	0	1 (0·4)
Delftia acidovorans	0	1 (0·7)	0	0	0	1 (0·4)
Exiguobacterium aurantiacum	0	2 (1·4)	0	0	0	2 (0·9)
Proteus mirabilis	0	1 (0·7)	0	0	0	1 (0·4)
Stenotrophomonas maltophilia	1 (8·3)	0	1 (4·5)	0	0	2 (0·9)
Ralstonia pickettii	0	0	1 (4·5)	0	0	1 (0·4)
Sphingomonas paucimobilis	0	1 (0·7)	0	0	0	1 (0·4)
**Gram-positive cocci**						
Staphylococcus aureus	2 (16·7)	7 (4·9)	0	5 (14·3)	1 (7·7)	15 (6·7)
Coagulase-negative staphylococci	3 (25·0)	9 (6·3)	1 (4·5)	10 (28·6)	8 (61.5)	31 (13·9)
Streptococcus pyogenes (GAS)	0	1 (0·7)	1 (4·5)	0	0	2 (0·9)
Streptococcus agalactiae (GBS)	1 (8·3)	1 (0·7)	0	0	0	2 (0·9)
Streptococcus spp non-A non-B	0	5 (3·5)	1 (4·5)	0	0	6 (2·7)
Enterococcus faecalis	1 (8·3)	0	1 (4·5)	0	0	2 (0·9)
Enterococcus faecium	0	1 (0·7)	0	3 (8·6)	0	4 (1·8)
**Fungi**						
Candida orthopsilosis	0	2 (1·4)	0	0	0	2 (0·9)
Candida tropicalis	0	2 (1·4)	0	0	1 (7·7)	3 (1·3)

Data are n (%).
GAS=Group A Streptococci; GBS=Group B Streptococci.

Supplementary table 9: Pathogen profile among INBORN vs. OUTBORN neonates

Pathogens	Inborn (n=85)	Outborn (n=138)
Klebsiella spp	22 (25·9)	31 (22·5)
E coli	10 (11·8)	23 (16·7)
CoNS	16 (18·8)	15 (10·9)
Enterobacter spp	10 (11·8)	16 (11·6)
Serratia marcescens	6 (7·1)	10 (7·2)
Staphylococcus aureus	5 (5·9)	10 (7·2)
Acinetobacter spp	5 (5·9)	4 (2·9)
Streptococcus spp	2 (2·4)	8 (5·8)
Enterococcus spp	3 (3·6)	3 (2·2)
Candida spp	2 (2·4)	3 (2·2)
Elizabethkingia spp	2 (2·4)	3 (2·2)
Pseudomonas spp	1 (1·2)	2 (1·4)
Others	1 (1·2)	10 (7·2)

Data are n (%).
CoNS=coagulase-negative staphylococci.

Supplementary table 10: Pathogen profile based on onset of sepsis

Pathogens	Early-onset (n=129)*	Late-onset (n=94)
Klebsiella spp	31 (25·0)	22 (24·7)
E coli	20 (16·1)	13 (14·6)
Enterobacter spp	17 (13·7)	9 (10·1)
Serratia marcescens	13 (10·4)	3 (3·4)
Acinetobacter spp	6 (4·8)	3 (3·4)
Elizabethkingia spp	1 (0·8)	4 (4·4)
Pseudomonas	1 (0·8)	2 (2·2)
Streptococcus spp	6 (4·8)	4 (4·4)
Enterococcus spp	2 (1·6)	4 (4·4)
Staphylococcus aureus	4 (3·2)	11 (12·4)
CoNS	19 (15·3)	12 (13·4)
Candida spp	1 (0·8)	4 (4·4)
Others	8 (6·5)	3 (3·4)

Data are n (%).
CoNS= coagulase-negative staphylococci
**The number includes a few pathogens isolated after day 3 of life. They are still included under early-onset sepsis because these neonates had onset of signs within the first 3 days of life but the cultures done later during the same episode turned out to be positive.*

Supplementary figure 3: Pathogen profile based on the day of isolation among neonates with culture-positive sepsis (n=223)

3A: Pathogen profile among all enrolled neonates

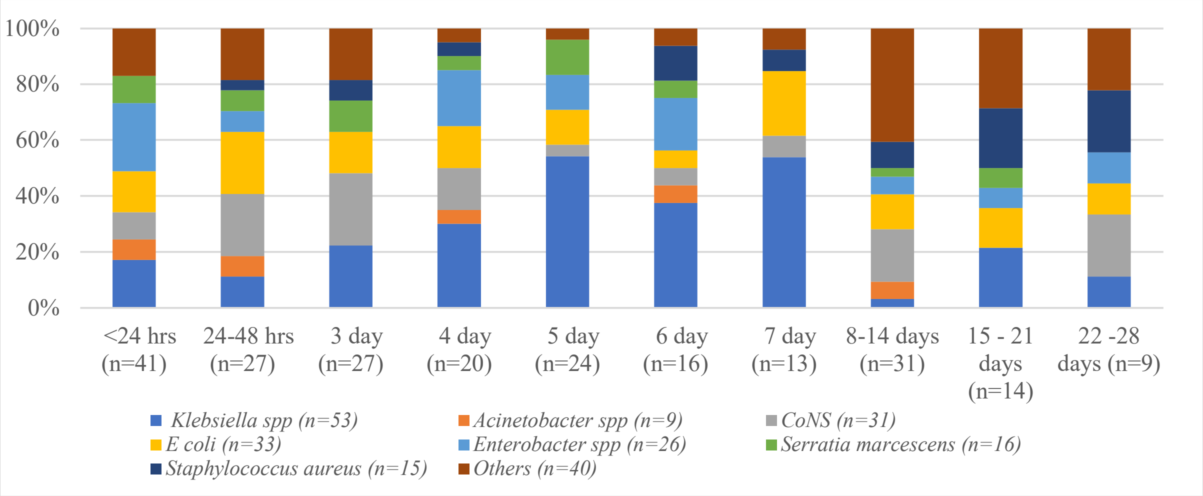

3B: Pathogen profile among INBORN neonates (n=85)

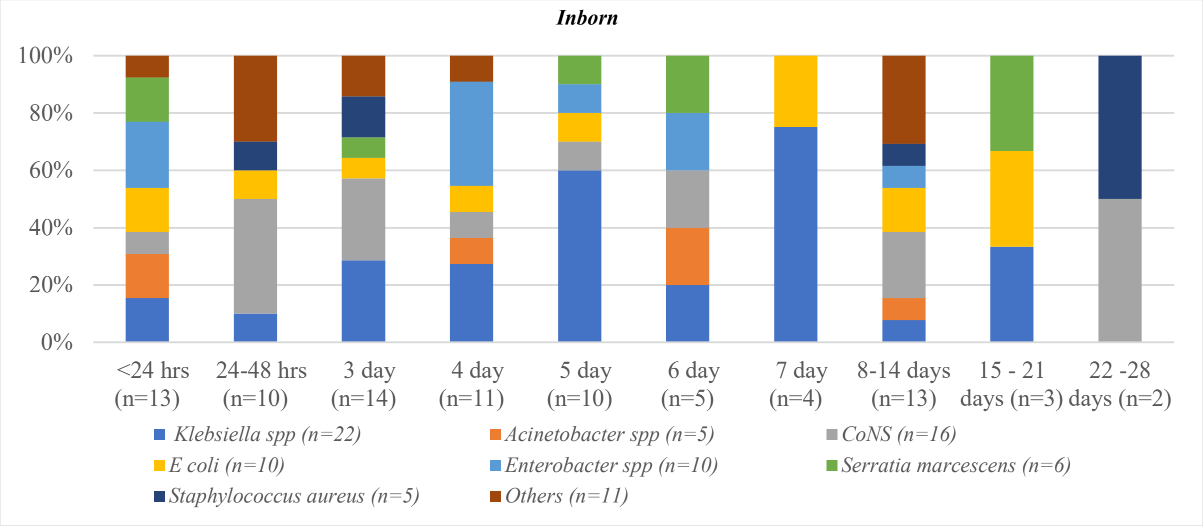

3C: Pathogen profile among OUTBORN neonates (n=138)

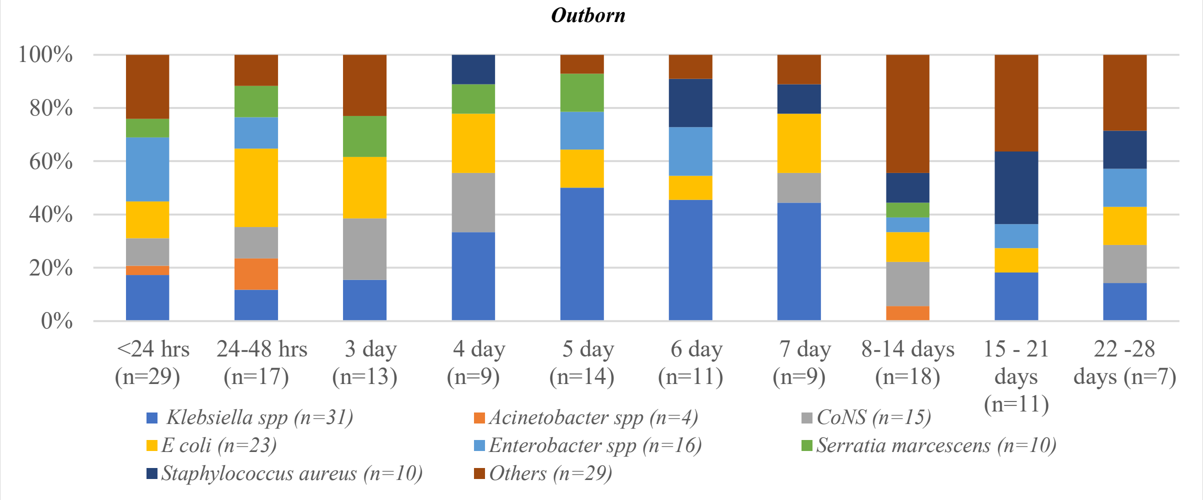

The figure depicts the number of pathogens isolated on a particular day irrespective of whether the onset was classified as early- or late-onset sepsis

Supplementary table 11: Pathogen profile among home deliveries

Pathogens	n=18
Escherichia coli	4 (22·2)
Staphylococcus aureus	3 (16·7)
Enterobacter spp	3 (16·7)
CoNS	3 (16·7)
Klebsiella spp	2 (11·1)
Streptococcus spp	2 (11·1)
Candida spp	1 (5·6)

Data are n (%).
CoNS= coagulase-negative staphylococci.

Supplementary table 12: Antimicrobial resistance pattern of common Gram-negative pathogens

Pathogens	*Klebsiella spp.* *(n=53)*	*E coli* *(n=33)*	*Enterobacter spp.* *(n=26)*	*Acinetobacter spp.* *(n=9)*	*Serratia marcescens* *(n= 16)*
Co-amoxiclav	38/52 (73·1)	10/32 (31·2)	-	0	1/2 (50)
Piperacillin+ tazobactam	26/52 (50)	6 (18·2)	10/25 (40)	7/8 (87·5)	3/7 (42·8)
Ciprofloxacin	29/52 (55·7)	25 (75·8)	18 (69·2)	6 (66·7)	11 (68·7)
Amikacin	21/51 (41·2)	4 (12·1)	6 (23·1)	6/8 (75)	2/15 (13·3)
Gentamicin	30/52 (57·7)	10 (30·3)	14 (53·8)	6 (66·7)	0
Cefoperazone + sulbactam	27/52 (51·9)	5 (15·1)	10 (38·4)	6 (66·7)	2/15 (13·3)
Ceftriaxone	43 (81·1)	25 (75·8)	21/25 (84)	5/7 (71·4)	2/15 (13·3)
Cefepime	21/52 (40·4)	11 (33·3)	1/25 (4)	7/9 (77·7)	-
Ceftazidime	18 (34)	13 (39·4)	13 (50)	6/9 (66·7)	0/15
Ciprofloxacin	29/52 (55·7)	25 (75·8)	18 (69·2)	6 (66·7)	11(68·7)
Imipenem	27 (50·9)	4 (12·1)	10 (38·5)	7 (77·7)	0
Meropenem	26 (49·0)	4 (12·1)	9 (34·6)	7 (77·7)	0/10
Tigecycline	2/42 (4·7)	0/32	2 (7·7)	0	11 (68·7)
Colistin	4/51 (7·8)	0	1/24 (4·2)	0	-

Data are n/N (%).
The denominator varies for some antibiotics because the antibiotic sensitivity testing could not be done for all isolates.

Supplementary table 13: Antimicrobial resistance pattern of common Gram-positive pathogens

Pathogens	*CoNS (n = 31)*	*S aureus* (n= 15)	*Streptococcus spp.* (n = 10)*	*Enterococcus spp. (n = 6)*
Penicillin	27/30 (90)	13/14 (92·8)	0/10	3/6 (50)
Cefoxitin	17/28 (60·7)	4/14 (28·6)	0/1	-
Ciprofloxacin	13/31 (41·9)	11/14 (78·6)	0/1	4/6 (66·6)
Gentamicin	5/28 (17·8)	3/14 (21·4)	0/3	2/3 (66·6)
Trimethoprim/sulfamethoxazole	10/29 (34·5)	4/13 (30·7)	3/4 (75·0)	0/1
Clindamycin	8/31 (25·8)	6/15 (40·0)	0/8	0/6
Erythromycin	16/29 (55·2)	10/14 (71·4)	3/7 (42·8)	5/6 (83·3)
Rifampicin	4 /31 (12·9)	0/12	0/8	0/6
Vancomycin	0/30	0/14	0/2	1/6 (16·6)
Teicoplanin	2/27 (7·4)	0/13	0/1	0/6

Data are n/N (%).
CoNS=coagulase-negative staphylococci; cefoxitin represents methicillin resistance
The denominator varies for some antibiotics because the antibiotic sensitivity testing could not be done for all isolates.
**Streptococcus agalactiae (n=2), Streptococcus galolyticus (n=1), Streptococcus pyogenes (n=2), Streptococcus sanguinis (n=3), Streptococcus viridans (n=1), and Streptococcus non-A non-B (n=1).*

Supplementary table 14: Antimicrobial resistance (AMR) and case-fatality rates by AMR pattern

Pathogens<br>Antimicrobial class	Resistant isolates*	Case-fatality rate	CFR in culture-positive sepsis due to	
			Sensitive pathogens	Resistant pathogens
Klebsiella spp. (n=53)	-	17/53 (32·1)	-	-
• Aminoglycosides	31/53 (58.5)	-	2/22 (9.1)	15/31 (48·4)
• 3rd gen cephalosporin	45/53 (84·9)	-	1/8 (12.5)	16/45 (35·6)
• Carbapenems	28/53 (52·8)	-	4/25 (16.0)	13/28 (46·4)
• BLBLI	40/53 (75.5)	-	2/13 (15.4)	15/40 (37.5)
• MDR	43/53 (81·1)		2/10 (20.0)	15/43 (34·9)
Escherichia coli (n=33)	-	17/33 (51·5)	-	-
• Aminoglycosides	11/33 (33.3)	-	13/22 (59·1)	4/11 (36.4)
• 3rd gen cephalosporin	26/33 (78·8)	-	6/7 (85.7)	11/26 (42.3)
• Carbapenems	4/33 (12·1)	-	16/29 (55·2)	1/4 (25·0)
• BLBLI	10/33 (30.3)	-	13/23 (56.5)	4/10 (40.0)
• MDR	28/33 (84·8)		4/5 (80.0)	13/28 (46·4)
Enterobacter spp. (n=26)	-	11/26 (42·3)	-	-
• Aminoglycosides	14/26 (53.8)	-	7/ 12 (58.3)	4/14 (28·6)
• 3rd gen cephalosporin	23/26 (88.5)	-	2/3 (66.7)	9/23 (39.1)
• Carbapenems	10/26 (38·5)	-	9/15 (60·0)	2/11(18.2)
• BLBLI	25/26 (96.2)	-	1/1 (100)	10/25 (40.0)
• MDR	23/26 (88·5)		2/3 (66.7)	9/23 (39·1)
Acinetobacter spp. (n=9)	-	2/9 (22·2)	-	-
• Aminoglycosides	6/9 (66.7)	-	1/3 (33.3)	1/6 (16·7)
• 3rd gen cephalosporin	6/9 (66.7)	-	1/3 (33.3)	1/6 (16·7)
• Carbapenems	7/9 (77·8)	-	1/2 (50·0)	1/7 (14.3)
• BLBLI	8/9 (88.9)	-	1/1 (100)	1/8 (12.5)
• MDR	7/9 (77·8)		1/2 (50·0)	1/7 (14·3)
Staphylococcus aureus (n=15)		2/15 (13·3)	-	-
• Ciprofloxacin	11/14 (78.6)		0/3 (0)	2/11 (18.2)
• Cefoxitin	4/14 (28·6)	-	1/10 (10·0)	1/4 (25·0)
• Vancomycin	0/14 (0)	-	2/14 (14·3)	0
CoNS (n=31)	-	6/31 (19·4)	-	-
• Ciprofloxacin	13/30 (43.3)	-	5/17 (29.4)	1/13 (7.7)
• Cefoxitin	17/28 (60·7)	-	3/11 (27·3)	1/17 (5·9)
• Vancomycin	0/30 (0)		6/30 (20·0)	0
Streptococcus (n=10)	-	5/10 (50·0)	-	-
• Ciprofloxacin	1/3 (33.3)	-	1/2 (50.0)	0/1 (0)
• Cefoxitin	0/4 (0)		2/4 (50·0)	0
• Vancomycin	0/10 (0)		5/10 (50.0)	0
Enterococcus faecium (n= 6)	-	1/6 (16·7)	-	-
• Ciprofloxacin	4/6 (66.7)	-	0/2 (0)	1/4 (25.0)
• Cefoxitin	-		-	-
• Vancomycin	1/6 (16·7)		1/5 (20.0)	0/1 (0)

Data are n/N (%).
BLBLI=β-lactam/β-lactamase inhibitors; CoNS=coagulase-negative staphylococci; MDR=multidrug resistant.
**Gram-negative pathogens were classified based on their resistance (intermediate or resistant) to the following antibiotic classes: Aminoglycosides (any one of gentamicin, amikacin, or netilmicin); 3rd generation cephalosporins (any one of cefotaxime, ceftriaxone, or ceftazidime); carbapenems (any one of imipenem, meropenem or ertapenem); β-lactam/β-lactamase inhibitors (BLBLI- any one of amoxicillin-clavulanic acid, piperacillin-tazobactam, or cefoperazone-sulbactam). Multidrug resistance (MDR) was defined as resistance to one or more agents in at least three antibiotic classes (3rd generation cephalosporins, carbapenems, aminoglycosides, fluoroquinolones, BLBLI, and polymyxins).*

Supplementary table 15: Incidence and case-fatality rate (CFR) of culture-negative sepsis

	**Incidence***	**Case-fatality rates†**
Overall	2076/6612 (31·4; 20·4-45·0)‡	88/2076 (4·4; 1·6-11·0)‡
Site 1	454/1742 (26·1; 24·0-28·2)	10/454 (2·2; 1·1-4·1)
Site 2	609/1358 (44·8; 42·2-47·5)	50/609 (8·2; 6·2-10·6)
Site 3	407/1020 (39·9; 36·9-43·0)	14/407 (3·4; 2·0-5·8)
Site 4	311/1100 (38·3; 25·6-31·0)	5/311 (1·6; 0·6-3·9)
Site 5	295/1392 (21·2; 19·1-23·4)	9/295 (3·1; 1·5-5·9)

**Data are number of cases (%; 95% CI). †Data are number of deaths/number of cases (%).*
NB: Case-fatality rate was calculated by dividing the number of deaths due to sepsis within 28 days of life or 21 days of suspicion of sepsis, whichever is earlier, by the number of neonates in the respective sepsis category.
‡The confidence interval is inflated, accounting for clustering within sites. Adjusted for cluster.

Supplementary table 16: Sepsis categories and duration of antibiotic therapy

**Categories**	**Duration of antibiotics (days)**	
	All neonates	After excluding deaths
Suspected sepsis but not classified as either culture-negative or culture-positive sepsis (n= 1068)	4 (3 to 5)	4 (3 to 5)
Culture-negative sepsis (n= 2076)	6 (2 to 7)	6 (5 to 8)
Culture-positive sepsis (n= 213)	6 (2 to 11)	8 (5 to 14)

Data are median (IQR).

Supplementary table 17: Neonatal mortality (all-cause death by 28 days of age) by place of birth: site-wise

	Overall deaths	Deaths among inborn	Deaths among outborn
**All sites***	**676/6612 (10·2; 2·4-35.0)**	**234/3972 (5·9; 1·9-17.0)**	**442/2640 (16·7; 4·9-44.0)**
Site 1	48/1742 (2.8; 2·1-3.7)	34/1421 (2.4; 1·7-3.4)	14/321 (4.4; 2·5-7.4)
Site 2	389/1358 (28.6; 26·3-31.1)	72/314 (22.9; 18·4-28.1)	317/1044 (30.4; 27.6-33.3)
Site 3	124/1020 (12.2; 10·2-14.4)	56/460 (12.2; 9·4-15.6)	68/560 (12.1; 9·6-15.2)
Site 4	50/1100 (4.5; 3·4-6.0)	33/903 (3.7; 2·6-5.2)	17/197 (8.6; 5·2-13.9)
Site 5	65/1392 (4.7; 3·6-6.0)	39/874 (4.5; 3·2-6.1)	26/518 (5.0; 3·4-7.4)

Data are n (%).

**The confidence intervals are inflated, accounting for clustering within sites.*

Supplementary table 18: Single most important underlying cause of death (until 28 days of life)

Causes of death	Overall (n=649/676)*	Inborn (n= 226/234)*	Outborn (n= 423/442)*
Prematurity	166 (25·6)	73 (32·3)	93 (22·0)
Asphyxia	215 (33·1)	68 (30·1)	147 (34·8)
Sepsis	179 (27·6)	48 (21·2)	131 (31·0)
Congenital malformations	17 (2·6)	7 (3·1)	10 (2·4)
Others	72 (11·1)	30 (13·3)	42 (9·9)

Data are n (%).
**Cause of death was not available for 27 neonates (8 inborn and 19 outborn).*

Supplementary table 19: Sepsis categories and the risk of mortality

	Died (n=676)	Survived (n=5936)	Hazard ratio (95% CI)	Adjusted hazard ratio* (95% CI)
Never suspected of sepsis (n=3255)	97 (3.0)	3158 (97.0)	-	-
Suspected sepsis but not classified as either culture-negative or culture-positive sepsis (n= 1068)	86 (8.1)	982 (91.9)	2·8 (1·2 to 6·2)	1·9 (0·98 to 3·7)
Culture-negative sepsis (n= 2076)	407 (19·6)	1669 (80·4)	7·2 (4·0 to 13.0)	4·6 (2·8 to 7·5)
Culture-positive sepsis (n= 213)	86 (40.4)	127 (59.6)	16·3 (8·6 to 31·2)	7·4 (4·7 to 11·6)

Data are n (%). The confidence intervals are inflated, accounting for clustering within sites.
Culture-positive sepsis vs culture-negative sepsis: adjusted hazard ratio is 1.8 (1.4 to 2.2)
**Adjusted for birth weight, gestation, major malformations, and asphyxia (did not cry at birth).*

Annexure 2

Burden of multidrug-resistant neonatal sepsis in district hospital settings in India

Standard Operating Procedure (SOP)

Version: 1.0, dated 18 Sep 2019

Research team

Details	Name & designation	Address	Email ID	Mobile no
**All India Institute of Medical Sciences (AIIMS), New Delhi: Coordinating site**				
Principal Investigator (PI)	Dr M Jeeva Sankar<br>Assistant Professor	Department of Pediatrics	jeevasankar@gmail.com	9818398928
Co-PI	Dr Ramesh Agarwal<br>Professor	Department of Pediatrics	ra.aiims@gmail.com	9810756718
Co-PI	Dr Kajal Jain<br>Scientist-II	Department of Pediatrics	kajaljain12@gmail.com	9911094787
Co-PI	Dr Arti Kapil<br>Professor	Department of Microbiology	akapilmicro@gmail.com	9868397951
Co-PI	Dr Sarita Mohapatra<br>Assistant Professor	Department of Microbiology	drsarita2005@gmail.com	9810726220
**Jawaharlal Institute of Postgraduate Medical Education and Research (JIPMER)**				
Principal investigator (Pediatrics; tertiary care hospital)	Dr. Nishad Plakkal<br>Associate Professor	Department of Neonatology, JIPMER, Puducherry 605006	plakkal@gmail.com	7708577133
Co-PI (Microbiology; Tertiary care hospital)	Dr. Apurba Sastry<br>Associate Professor	Department of Microbiology, JIPMER, Puducherry 605006	drapurbasastry@gmail.com	9444327314
Other Co-PI	Dr. B. Adhisivam, Additional Professor<br>Dr. Sindhu Sivanandan, Assistant Professor	Department of Neonatology, JIPMER, Puducherry 605006	adhisivam1975@yahoo.co.uk<br><br>drsindhusivanandan@gmail.com	9488822113<br><br>9968935020
**Government Headquarters Hospital, Cuddalore, Hospital Rd, Manjakuppam, Cuddalore, Tamil Nadu 607001**				
Co-I (DH)	Dr. K. M. Madhan Kumar<br>Assistant Surgeon	Department of Pediatrics, Govt. Headquarters Hospital, Cuddalore,<br>Tamil Nadu 607001	madhankidsdoc@gmail.com	9884519845
Co-I	Dr. N. Hafiza<br>Hospital Superintendent	Govt. Headquarters Hospital, Cuddalore, Tamil Nadu 607001	cuddaloregh@gmail.com	9445230749
**All India Institute of Medical Sciences (AIIMS), Raipur**				
PI	Dr. Atul Jindal<br>Associate Professor	Department of Pediatrics, AIIMS, Raipur	dratuljindal@gmail.com	8224014667

Co-PI	Dr Anudita Bhargava, Additional Professor	Department of Microbiology, AIIMS, Raipur	anuditabhargava@gmail.com	8518881903
**Mahasamund District Hospital, Kharora, Chhattisgarh 493445**				
Co-I	Dr. Prabir Chatterjee Executive Director	SHRC, Chhattisgarh	prabirkc@yahoo.com	7583031009
Co-I	Dr Amar Singh Thakur Deputy Director, State Immunization	Deputy Director (Child Health), Directorate Health Services, third floor, Indrawati Bhawan, Atal Nagar, Raipur, Chhattisgarh	amarcmo@gmail.com; ddchildhealth.cg@gmail.com	9425219353
Co-I	Dr Harish Bagh	Mahasamund District Hospital, Kharora, Chhattisgarh 493445	harishbagh1215@gmail.com	8085164865
**Government Medical College and Hospital (GMCH), Chandigarh**				
PI	Dr Deepak Chawla	Department of Neonatology, GMCH, Chandigarh	drdeepakchawla@gmail.com	9646121559
Co-PI	Dr Suksham Jain	Department of Neonatology, GMCH, Chandigarh	drsukshamj@gmail.com	9988901596
Co-PI (Microbiology)	Dr Jagdish Chander	Department of Microbiology, GMCH, Chandigarh	jchander@hotmail.com	9646121570
	Dr Nidhi Singla	Department of Microbiology, GMCH, Chandigarh	nidhi0402@hotmail.com	9646121586
**Regional Hospital, Hamirpur Road, Una, Himachal Pradesh 174303**				
Co-I	Dr Ravi Sharma	District Hospital, Una	drravikudini@yahoo.co.in	9418485188
Co-I	Dr Mangla Sood	National Health Mission, HP	drmanglasood@gmail.com	9418453465
**Assam Medical College, Dibrugarh**				
PI	Dr Reeta Bora	Neonatology Unit, Dept of Pediatrics, AMCH	bora64reeta@gmail.com	9435394313
Co-PI	Dr Aukifa K S Islam	Neonatology Unit, Dept of Pediatrics, AMCH	aukifa.islam@yahoo.co.in	9435033510
Co-PI (Microbiology)	Dr Reema Nath, Dr Partha Pratim Das	Dept of Microbiology, AMCH	reema_44rediffmail.com, drppd83@gmail.com	9435031619, 9864300000
**Sivasagar Civil Hospital, Joysagar, Rupahi Pathar, Assam 785665**				
Co-I	Dr Sukalyan Das	District Hospital, Sivasagar	drdassukalyan@gmail.com	9435056670

**All India Institute of Medical Sciences (AIIMS), Jodhpur**				
PI	Dr Neeraj Gupta, Associate Professor	Department of Neonatology	neerajpgi@yahoo.co.in	8003996908
Co-PI (Clinical)	Dr Kuldeep Singh; Professor & HOD	Department of Pediatrics	kulpra@gmail.com, singhk@aiimsjodhpur.edu.in	8003996940
Co-PI (Microbiology)	Dr Vijay Lakshmi Nag; Professor & HOD	Department of Microbiology	nagv@aiimsjodhpur.edu.in	8003996874
Co-PI (Microbiology)	Dr Anuradha Sharma, Additional Professor	Department of Microbiology	sharmaa@aiimsjodhpur.edu.in	8003996896
**Government Nahata Hospital, Government Nahata Hospital Road, Balotra, Rajasthan 344022**				
Co-I (Clinical)	Dr Kamal Kishore Mundra	Senior Specialist (Pediatrics)	drkamal70@gmail.com	9414384046
Co-I (Admin)	Dr Balraj Singh Panwar	Principal Medical Officer	dr.balrajsinghpanwar@gmail.com	9460537979
Co-I (Clinical)	Dr Dhannaram Gossai	Junior Specialist (Pediatrics)	drg1058@gmail.com	9828655075
**Clinical Development Services Agency (CDSA)**				
	Ms Shubhra Bansal	Director – Clinical Portfolio Management	shubhrabansal.cdsa@thsti.res.in	9810709126
	Mr Prashant Bhujbal	Manager- Admin and Finance	prashantbhujbal.cdsa@thsti.res.in	8130971258
	Ms Preeti Semwal	Project Manager	preetisemwal.cdsa@thsti.res.in	9937411885
	Mr Danish Nafees	Study Monitor	danishnafees.cdas@thsti.res.in	9891768614
	Mr Girish Mishra	Study Monitor	girishmishra.cdsa@thsti.res.in	8010803421
	Mr Narendra Singh	Assistant Data Manager	narendrasingh.cdsa@thsti.res.in	6392121019

Contents

	Page no.
**Project summary**	
**Part I: Clinical SOP**	
Overview of the study	
Roles and responsibilities	
Quality control	
**Part II: Microbiology SOP**	
Roles and responsibilities	
Collection of samples at District Hospital (DH)<br>Transport of samples from DH to tertiary sites<br>Processing of samples at tertiary site	
Antimicrobial susceptibility testing	
Biochemical tests	
Preservation of isolates	
Transport of isolates to the reference laboratory	
Quality control	
Annexure 1 - Blood culture	
Annexure 2 - Sepsis screen	
Annexure 3 - Lumbar puncture	

Part 1 – Clinical SOP

- Overview of the study
- Roles and responsibilities
- Quality control

Project summary

Background

Infections are the major cause of deaths in the neonatal period. In our recent study, *Acinetobacter spp., Klebsiella spp., Escherichia coli* and *Staphylococcus aureus* together constituted ~ 65% of EOS burden with Acinetobacter spp., emerging as the predominant one (DeNIS). The available data and pointers attest to a dangerous trend toward antimicrobial resistance among the common strains.

Additionally, majority (68%) of sepsis cases were EOS (< 72 h), associated with multidrug-resistant (MDR) pathogens, and resulted in high mortality (of up to 59%). This dominance of "nosocomial-type", LOS-causing bacterial pathogens in EOS suggests that, in our settings, pathogens causing EOS may be acquired through horizontal transmission perhaps from delivery rooms and/or NICUs within 72 h of birth. As data on neonatal sepsis in secondary hospitals is not available, we will perform an observational study in five neonatal units across secondary/district hospitals and collect high-quality observational data on prevalence of sepsis burden, pathogens causing the burden and their AMR status.

Aim

To determine the sepsis burden, pathogens responsible and their antimicrobial resistance profiles among neonates admitted in secondary care settings

Objectives

1) To determine the burden of sepsis and sepsis-related mortality among admitted neonates
2) To identify the predominant bacterial pathogens that cause early and late-onset neonatal sepsis
3) To determine the antimicrobial resistance profiles of the isolated pathogens

Study design

Prospective cohort study

Setting

Level II special newborn care units (SNCU) of district hospitals under supervision of tertiary sites

Method

This prospective study would be conducted in five district hospitals of India. All neonates (inborn and outborn; postnatal age less than or equal to 28 days) admitted in the SNCUs of the district hospital sites for any indication during the study period of 18 to 21 months will be tracked by the research staff and enrolled after taking consent from the parents.

Eligibility criteria

Inclusion criteria: All neonates (inborn and outborn) admitted in the SNCUs of the district hospitals; postnatal age less than or equal to 28 days

Exclusion criteria: Parents not willing to participate in the study

A dedicated team of research nurses at each site will track all the babies admitted in SNCU until 28 days of age or death. In neonates with suspected sepsis (based on definitions provided in next section), sepsis work-up including cultures of blood and other body fluids will be performed as per the Standard Operating Procedures (SOP). For each suspected episode, after reviewing the clinical course, sepsis screen, and culture reports, the assigned pediatrician at each site would make a final diagnosis of sepsis or 'no sepsis'. Samples for culture will be sent to microbiology lab for characterization of bacterial isolates by automated culture system and determination of antibiotic susceptibility pattern. Strict quality assurance measures covering clinical, microbiology, and data management will be implemented at all sites. Isolates will be stored for whole genome sequencing.

Clinical and microbiology data will be collected in source forms. Data will be entered by the DEO in eCRF. The data would be continuously checked by the SRN, DH co-Investigator and PI. The data would be cross-checked by the study monitor (during the visit to the site) and verified with source documents.

Study deliverables

The study shall provide data on the following outcomes:

- Incidence of early onset sepsis and late onset sepsis, and culture positive and culture negative sepsis (inborn neonates)
- Prevalence of culture positive and culture negative sepsis (Out born neonates)
- Mortality – all cause as well as sepsis-related mortality
- Profile of organisms isolated
- Antimicrobial resistance pattern
- Details of antibiotic therapy
- Molecular characterization of virulence factors and resistance genes

Definitions

Definition of different terms	
**Enrolled**	All neonates admitted to SNCU of ≤28 days of age with consent
**'Suspected sepsis'**	Age of the neonate ≤28 days<br><br>AND<br><br>Presence of any one of the risk factors/ clinical symptoms/signs from the following list, for which the neonate has no other known or reliable explanation (*to be verified independently by a neonatologist from the corresponding tertiary site later)*:<br><br>***Perinatal risk factors***<br><br>1. Foul-smelling liquor<br>2. At least two of the following: (i) spontaneous prematurity and preterm pre-labor rupture of membranes (PPROM; irrespective of duration of rupture of membranes) (ii) Febrile illness in the mother with suspected bacterial infection warranting start of antibiotics by attending clinician in the week prior to delivery (iii) rupture of membranes >24 hours (iv) unclean vaginal examination(s) during labor<br><br>***Clinical symptoms/signs***<br>3. No movement or movement only when stimulated<br>4. Refusal to feed<br>5. Severe chest in-drawing or increased oxygen requirement or need for respiratory support<br>6. Grunting<br>7. New onset apnea or increased severity or frequency of apnea in a baby who already is having apneas<br>8. Cyanosis or desaturation needing oxygen therapy or respiratory support (or increase in oxygen requirement/deterioration in a baby who is already receiving oxygen therapy)<br>9. Fever or hypothermia (>37.5°C or <36.5°C)<br>10. Tachycardia or episodes of bradycardia (>180/min or <100/min)<br>11. Capillary refill time (CRT) > 3 sec<br>12. Mottled skin or other evidence of shock<br>13. Erythema in the skin around umbilical stump (extending to >1 cm)<br>14. Lethargy or drowsiness<br>15. Convulsions<br>16. Abnormal posturing<br>17. Hypotonia or floppiness<br>18. Bulging fontanelle<br>19. Vomiting or abdominal distension<br>20. Bleeding<br>21. Sclerema
**Culture-positive sepsis**	Neonate with 'suspected sepsis' (see above) AND isolation of a recognized pathogen from blood, cerebrospinal fluid, or other body fluids, along with intended treatment of at least 5 days of antibiotics

**Culture-negative sepsis**	Neonate with 'suspected sepsis' AND negative blood culture (or blood culture deemed to have grown a commensal) AND one or more of the following:<br>1. Clinical course consistent with sepsis and there was no alternative explanation for the clinical symptom(s)<br>2. Positive laboratory criteria (at least one of the following three: white blood cells count <4.0 x 10^9 cells/L; absolute neutrophil count < 1.5 X 10^9 cells/L; C-reactive protein (CRP) > 6mg/L)
**No sepsis**	Neonates fulfilling one of the following criteria:<br>1. Never met 'suspected sepsis' criteria until 28 days of age (i.e. never subjected to blood culture or laboratory tests)<br>2. With 'suspected sepsis' but negative blood culture report and did not meet criteria for culture negative sepsis (above)
**Early-onset sepsis**	Occurrence (i.e. time of onset of symptoms) of culture-positive or culture-negative sepsis at or before 72 h of life
**Late-onset sepsis**	Occurrence (i.e. time of onset of symptoms) of culture-positive or culture-negative sepsis after 72 h of life
**Meningitis**	Positive cerebrospinal fluid culture, Gram staining, or neutrophilic leukocytosis, with or without low glucose (<50% of plasma glucose level) and high protein content<br>(Normal range of CSF components: cells - up to 30 cells/mm^3; polymorphonuclear leukocytes (PMN) - 60%; protein: up to 150 mg/dL; CSF/blood glucose >60%
**Systemic fungal infection**	Blood culture, CSF, or suprapubic urine positive for yeasts or presence of budding yeast/hyphae AND<br>physician institutes appropriate therapy for fungal infection
**New episode of sepsis**	When the neonate become symptomatic after 48 hours of stopping appropriate antibiotic therapy, or if a new organism is cultured, with fresh clinical deterioration during an ongoing episode.

Overview of roles and responsibilities of DH, tertiary sites, and nodal site

District hospital →

- Follow-up of all the neonates admitted in SNCU (enrolled neonates) till 28 days of life or until discharge or death; Form A - Part I and Monitoring sheet/ Form B)
- Sepsis work-up for neonates suspected of sepsis (Form A - Part II, Monitoring sheet / Form B)
- Sample collection for culture, placing the sample at room temperature or in incubator, and getting transported to tertiary site (Form C)
- Sample collection for laboratory parameters (WBC, ANC and CRP)
- Receiving culture report and final labelling of episode
- Completion of forms (Form A, B and C)
- Filling eCRF

Tertiary site →

- After receiving samples for culture, placing them in automated culture system for at least 5 days
- Sub-plate the sample with appropriate labelling before placing it in automated culture system, if the sample has been collected >=24 hours prior to putting it in automated culture system
- Identification of isolate and reporting AST using conventional/automated method (depending on availability at tertiary site)
- Storage of isolates and transport (in duplicate) to nodal centre
- Coordinating with clinical team and complete Form C
- Supervising and mentoring DH site
- [illegible]

Nodal centre (AIIMS, New Delhi) →

- Confirmation of identification (by MALDI-ToF) and AST (by VITEK)
- Storage of isolates for molecular work
- WGS of representative samples of Gram-negative isolates for resistance and virulence genes
- Coordinating with all the sites and CDSA

Overview of methods and responsibilities of research staff

Steps	By whom?
**Track all babies admitted in the SNCU in the last 24 hours**<br>• Individually track all babies (register)<br>• Collect baseline information and fill-up form A (part I)	**Research nurse;** supervised by senior research nurse **(SRN)**
Record daily if the baby has any signs of sepsis<br>*(Fill-up part II of Form A)*	**Research nurse;** supervised by **SRN/ DH Co-I**<br>All entries made by DEO to be checked by SRN
**If sepsis is suspected**	
**Investigations to rule out/rule-in sepsis**<br>• Blood/ CSF culture<br>• WBC, ANC and CRP, CSF biochemical analysis<br>• Others (e.g. chest X-ray) as indicated	**Resident/research nurse;** supervised by site PI/Co-PI (microbiology)<br>To be done and interpreted by the **resident/research nurse**
Labeling the samples (particularly blood culture bottle)	**Research nurse/resident** (whoever takes the blood sample); Supervised by **SRN**
Keeping the sample at room temperature	**Research nurse/resident** (whoever takes the blood sample); Supervised by **SRN**
Filling-up the requisition forms and Form C	Requisition form: **Research nurse**<br>Form C: **SRN**
Transport of samples along with the forms to the tertiary site laboratory	**Laboratory attendant;** supervised by **research nurse**
Collecting and filing the reports	**Research nurse;** supervised by **SRN**
Completing Form A and C based on the laboratory reports and case-files	Form A: **Research nurse**<br>Form C: **SRN/Lab Technician supervised by PI, Co-I (Micro)**
Handing over the filled and updated forms to DEO	Form A: **SRN**<br>Form C: **SRN/Lab technician / Co-I (micro)**
Data entry	**Data entry operator;** supervised by **site PI**

Detailed steps and responsibilities

Identification and enrolment

- Enrolment will be carried out 24x7 on all days (tertiary site PI and co-I at DH can decide if there is a need to have one free day, i.e. Sunday, based on the load of patients, workload of research nurse, and other local factors). If an RN is not available to cover a shift, events during that shift are to be covered by SRN or the RN on the next shift.
- RN should discuss with SRN and co-I (DH) before sampling, in case she suspects sepsis as per our study definitions
- The research nurse (RN) shall start tracking the new babies admitted in the SNCU during her shift time.
- The RN shall maintain a register to record the details of all neonates admitted in SNCU
- Senior Research Nurse (SRN) shall supervise enrolment of neonates daily; she shall counter-check the Log/register with that of the SNCU register to identify any missing enrolment
- Clinical management of all enrolled neonates (feeding, method of feeding – oral vs. parenteral, monitoring for complications other than sepsis, etc.) will be based on the units' existing protocol.

Baseline data collection

- RN will retrieve baseline information from the case files and fill-up the part-I of Form A; in case of any query, she will contact the medical officer/clinical nurse.
- SRN shall supervise data retrieval on a daily basis

Monitoring for sepsis

- A uniform protocol will be followed for screening the neonates for sepsis on the basis of clinical symptoms/signs (see above; page 8 and 9).
- RNs shall attend the SNCU rounds by the Consultant to collect information about babies suspected to have sepsis
- In addition, RN shall examine the neonates independently to assess for presence of symptoms/signs of sepsis; if identified, she will inform the DH co-Investigator or senior research nurse and confirm if the neonate does have features of sepsis
- RN shall update the daily monitoring form for sepsis every 24 hours, until the baby is shifted to the ward or discharged from the hospital.

For neonates suspected to have sepsis

- In neonates suspected to have sepsis (identified and/or confirmed by the Consultant or senior clinical nurse), RN will collect the blood samples for culture, laboratory parameters, and other investigations
- The RN shall ensure that the blood sampling for culture is done before initiation of antibiotics
- RN will inform the SRN before collecting the samples
- SRN will fill Form C and then send it to the microbiology team
- RN will help the medical officer /clinical nurse in emergency management like initiation of oxygen therapy, treatment of seizures, etc.

- Further clinical management including antibiotic therapy, ordering investigations like X-ray chest/abdomen, CSF examination etc. will be decided by the clinical team

Collection, labeling, and storage of blood/CSF samples

- Blood, CSF, and other cultures will be collected as per standard guidelines (see *Annexure*)
- RN will label the samples and place it in incubator set at 35°C, if the sample will reach tertiary site microbiology within 20 hrs of collecting the sample. Else the samples should be kept at ambient room temperature in SNCU corner away from sunlight ; the samples should NOT be refrigerated.
- CSF sample should be sent on the same day to the tertiary site's microbiology laboratory. The research nurse should co-ordinate with the laboratory attendant to ensure that the sample is sent to the laboratory immediately.
- Of the CSF collected, at least 1 mL should be loaded in the culture bottle and 5-6 loops should be put on a slide and smear is prepared. Other tests can be done by the clinical team based on local protocols.
- A uniform label will be used for all the samples at all sites. The concerned DEO/SRN will ensure that these labels are captured in the culture bottles before being sent to the laboratory. Label should have baby's name, Unique Identification number and date of collection of samples.
- Labelling will comprise of Unique Identification number of the neonate, type of sample, number of samples taken for a particular baby, and the date and time of sampling. For eg: If Unique Identification number of a baby is 50001 and first blood sample is taken for the baby, labelling shall be 50001-B1 and the date. For CSF, 'C' should be used instead of 'B'.

Unique Identification no:	50001-B1
Date of sampling:	30.08.2019
Time:	1430 hrs

- The RN will fill up the Form C (page 1) and hand over the samples along with Form C/Lab requisition form to the lab attendant once daily (at 11 AM – to be decided as per the laboratory timings of each study site)

Transport of samples and collecting the reports

- The laboratory attendant will transport the samples securely, using the container provided, from the DH to the microbiology laboratory of the tertiary site daily (except on Sundays) – at 11 AM
- The laboratory attendant will also handover the reports from the laboratory to RN/SRN
- The travel grant provided to the tertiary site will be utilized to pay for the local travel of the laboratory attendant by public transport.

Updating the proforma and handing over to DEO

- RN will update the Form A of each neonate daily until they are discharged from the hospital (or until their death)
- All enrolled neonates are to be followed-up until discharge from the hospital; if discharged before 28 days of age, a follow-up telephonic call/ visit has to be made by RN to complete the outcome at 28 days of age.
- RN will hand over the completed Form A to the DEO after getting countersigned by the SRN within 24 hours of discharge/death
- RN will maintain a register to keep record of the forms that are being sent to DEO; she shall get the signature of the DEO in the register for the forms that are dispatched to him/her
- SRN, during her weekly visit to tertiary site, will make sure that the Form C is filled up by laboratory technician/SRN (supervised by microbiology Co-I) and handed over to DEO
- *Quality check:* SRN will cross-check the forms filled by the nurse and give the feedback to the nurse
- All the completed forms should be counter-signed by the SRN and site PI/co-PI

Data entry

- The data entry operator (DEO) posted at DH will enter the data for a particular baby in the database for all the sections that are reviewed and signed by the DH co-Investigator (including babies who are still admitted).
- DEO shall maintain a register to keep record of the forms that are completed and entered. In case of queries, DEO should raise the queries to tertiary site PI.

Storage of samples and transport to nodal center

- Each microbiology laboratory will store the positive cultures in their respective laboratories; the isolates should be sent to the reference laboratory (AIIMS) once in three months.
- The isolates will be transported from the sites to AIIMS through courier service.
- External quality assessment: Identification and sensitivity of all the isolates from each centre will be confirmed at AIIMS reference centre through MALDI-TOF and VITEK 2 respectively.
- Isolates will be stored in glycerol stock for molecular characterization.

Part 2 – Microbiology SOP

- Transport of specimen from DH site to tertiary site
- Processing at the tertiary sites
- Preservation
- Transport of isolates from tertiary site to AIIMS, New Delhi
- Quality control

Roles and responsibilities

Role/responsibility	Person in-charge	Supervised by	Comments
**Transport of the samples (blood/CSF) to the laboratory**	Laboratory attendant	Research Nurse	Once during the day: Morning – between 10 and 11 AM
**Processing of samples (blood/CSF) in the laboratory including plating, making smears, subcultures, antibiotic sensitivity, and reporting**	Laboratory technician	Co-I (microbiology)	Daily as and when the sample is received
**Preparation of media, culture bottles, etc.**	Laboratory technician	Co-I (microbiology)	As and when required
**Replenishing the blood culture bottles in the SNCU**	Research Nurse	SRN	Daily
**Reporting**	Co-PI (Microbiology)	PI/Co-PI	Daily
**Maintaining the register/repository of all the culture reports**	Lab technician	PI/Co-PI (Microbiology)	Daily
**Keeping an inventory of the items (media, biochemical reagents, other consumables) received from the central supply**	Lab technician	PI/Co-PI (Microbiology)	As required
**Data entry (Form C)**	DEO	PI/Co PI (Microbiology)	Daily
**Transport of samples to reference laboratory (AIIMS)**	Laboratory technician	PI/Co PI (Microbiology)	Once in three months through courier services

Collection of samples at DH

Samples (blood, CSF) should be collected under aseptic conditions.

- In case of suspected sepsis (as per study definitions), blood sample should be collected under aseptic conditions (details in SOP video). At least 1mL blood sample should be loaded in the automated culture bottle RN will label the samples and place it in incubator set at 35°C, if the sample will reach tertiary site microbiology within 20 hrs of collecting the sample. Else the samples should be kept at ambient room temperature in SNCU corner away from sunlight; the samples should NOT be refrigerated.

Note:

• Up to 20 hours if vials are incubated* prior to entry in the instrument, or

• Up to 48 hours if vials are not incubated* (i.e., held at room temperature)

Cerebrospinal fluid (CSF) collection is not mandatory. It depends on clinician's discretion.

In case of CSF collection, it should be collected under aseptic conditions as per Annexure 2. At least 1 mL CSF should be loaded in the automated culture bottle, properly labeled and kept in the incubator set at 37°C.

For smear preparation: Centrifuge the CSF sample at 1500g for 10 min. Load 5-6 loops of CSF on the slide and air dry without spreading it. Label the slide and the slide should be stored at room temperature in a slide box.

CSF sample (in culture bottle and smear slide), if collected, should be transported to the tertiary site, the same day.

Note: Labelling will comprise of Unique Identification number of the neonate, type of sample, number of samples taken for a particular baby and the date of sampling. For eg: If Unique Identification number of a baby is 50001 and first blood sample is taken for the baby, labelling shall be 50001-B1 and the date. For CSF, 'C' should be used instead of 'B'.

Transport of samples from DH to respective tertiary site

The collected samples should be transported from the DH site to the tertiary centre earliest possible (within 24 hrs of collection of the sample).

Culture bottles should be kept in Sample carrier boxes (with proper cushioning by using thermocol, if required, to avoid breakage) and transported at room temperature.

Slides should be transported in slide box.

The sample(s) and form(s) should be handed over to the lab technician (microbiology department; tertiary site), on the same day.

Processing of samples at tertiary site

- Entry of received samples should be made in a register with date.

Culture bottles:

- The culture bottles should be checked for labelling and placed in the automated culture system. Time of loading the sample and other details should be noted in the Form 'C'. The samples should be placed in the automated culture machine for at least 5 days before considering negative. Culture bottles will be incubated in the automated system. The samples will be sub-cultured in the 5% sheep blood agar and MacConkey agar, if the delay in sample collected is >= 24 hours prior to putting it in automated culture system.

Smear slide: *Reporting smear results:* Pus cells/Oil Immersion Field and type of bacteria if present (morphology and gram stain)

Note: If smear positive: direct sensitivity may be put up; ***convey the preliminary report to the clinical team on telephone***

Identification and Antimicrobial susceptibility testing (AST) of the isolate

Identification of the isolate

Identification of the isolate should be done by automated system (VITEK 2). In case of absence of automated system, standard biochemical tests should be used.

Kindly look for the budding yeast cell colonies in the culture media to process further and corelate clinically.

Antimicrobial susceptibility testing (AST):

AST of the isolate should be checked by automated method (VITEK 2). In case of absence of automated method, conventional disk diffusion method should be used.

CLSI 2019 guidelines will be used to check AST for this year, CLSI 2020 for next year and CLSI 2021 for the following year.

Note:

The investigators from all the sites had detailed discussion and it was decided that blood agar from BioMerioux, McConkey and Mueller Hinton agar from Himedia will be obtained and used for this study.

VITEK 2 GN and GP cards will be used for identification of the isolates.

AST cards: AST 628, AST 280 and AST 281 will be used for the antibiotic susceptibility testing.

In case of fungal infection, VITEK 2 YST card will be used for identification and AST-YS08 should be used for antibiotic susceptibility testing.

If disc diffusion method is being used for AST, discs should be obtained from Biorad/ BD' company.

Antimicrobial susceptibility testing: Manual method

Preparation of Plates

Mueller-Hinton media is used. Defibrinated blood is necessary for testing fastidious organisms such as *Streptococcus, Enterococcus* etc. in which cases Muller Hinton plates supplemented with 5% sheep blood is used. pH of media is 7.2-7.4.

The medium is poured onto petri dishes to a depth of 4mm (Plate – 6 inches)

In the smaller plates up to 6 antibiotic discs are applied whereas in the larger plates up to 12 antibiotic discs are applied.

Poured plates are stored at 4^0C.

Before inoculation plates are dried by keeping in incubator at 37^0C with upside down and lid on agar so that there are no droplets of moisture for 30 minutes.

Preparation of Inoculum

One or two morphologically similar colonies were picked up by the straight wire and transferred to a test tube containing sterile normal saline. The density of the suspension is standardized by further dilution if necessary, to match the density visually with McFarland 0.5.

Antibiotic discs of Biorad/ BD will be used for antimicrobial susceptibility testing.

Inoculation

Plates are inoculated with a sterile cotton swab which is dipped into suspension of organism in normal saline and surplus removed by rotation of the swab against the side of the tube above the fluid level. The medium is inoculated by even streaking of the swab over the entire surface of the plate in several directions.

Antibiotic discs

After the inoculum has dried, single discs are applied with forceps. Discs are stored at 4^0C in sealed container and are allowed to come to room temperature before the containers are opened.

Incubation - Plates are incubated for 16-18hours at 37^0C.

Reading of Zones of Inhibition

The diameters of zones are measured to the nearest millimeter with a millimeter scale. The point of abrupt diminution of growth, which in more cases corresponds with the point of complete inhibition of growth is taken as the zone edge.

Interpretation

Each zone size is interpreted by reference table given by CLSI 2019 guideline to one of the following categories

Susceptible - infection treatable with normal dosage.

Intermediate/Partial- Infection that may respond to therapy with higher dosage or if infection is localized in a site where the agent is concentrated.

Resistant - not treatable with the agent.

For Gram positive organisms	***For Gram negative isolates***
1. Amoxycillin	1. Amoxycillin
2. Cefazolin	2. Co-amoxyclav
3. Co-amoxyclav	3. Cefotaxime
4. Ciprofloxacin	4. Ceftazidime
5. Gentamicin	5. Ceftriaxone
6. Amikacin	6. Gentamicin
7. Netilmycin	7. Amikacin
8. Vancomycin	8. Netilmycin
9. Teicoplanin	9. Ciprofloxacin
10. Linezolid	10. Piperacillin+tazobactam
11. Co-trimoxazole	11. Cefoperazone +Sulbactum
12. Clindamycin	12. Meropenem
13. Erythromycin	13. Imipenem
14. Tigecycline	14. Ertapenam
15. Cefoxitin	15. Doripenem
16. Penicillin	16. Tigecycline
17. Amoxclav	17. Colistin
18. Rifampicin	18. Cefipime
19. Chloramphenicol	20. Ampi-sulbactum

Mueller Hinton Agar (MHA) for sensitivity tests
Preparation of 0.5 McFarland Standards (Bailey and Scott, 1994)

0.05 ml of 1.175% (w/v) solution of barium chloride dehydrate ($Bacl_22H_2O$) (Hi Media Laboratories Limited, Mumbai, India) was added slowly and with constant agitation to 9.95 ml of 1% sulphuric acid (H_2SO_4) Merck (India) limited, Mumbai) to make a total volume of 10 ml in a 15 ml screw capped test tube. The tube was stored in dark at room temperature.

Reference strains for Quality Control of the Discs

Escherichia coli ATCC 25922, Pseudomonas aeruginosa ATCC 27853 and Staphylococcus aureus ATCC 25923 were used as reference strains for quality control and the results interpreted as per CLSI guidelines (2019).

Biochemical tests

Primary responsibility: **Laboratory technician (microbiology)** To be supervised by: **Co-PI**

Standard tests to be used for identification of bacteria - briefly,

Smear

↓

Gram Stain

Gram positive

Catalase test
Coagulase test
Bile esculin
6.5% NaCl,
Mannitol
Arabinose (E.faecalis -ve, E.faecium +ve)
Bile solubility test, optochin sensitivity test
Bacitracin sensitivity test
CAMP test

Gram negative

Catalase test
Oxidase test
Sugars for identification
Indole
Citrate
Urease
TSI
Motility testing

Preservation of isolates

Primary responsibility: Lab technician To be supervised by: **PI/Co-PI**

Isolates should be stored as stab stocks in Nutrient Agar at -20°C (in triplicate) with proper and complete labeling.

Labelling: Unique Identification number of the neonate, type of sample, serial number of the sample taken for a particular baby and the date of sampling. For the three tubes, A, B and C should be added at the end.

In case of more than one organism in the same culture, a symbol of '*' should be added.

Out of the three tubes, one stock tube should be retained at the site.

The other two stock tubes (in nutrient agar) should be transported to the nodal centre (AIIMS, New Delhi) through courier services at room temperature.

Steps for preserving the bacterial isolates

1. ***Stab stocks in Nutrient Agar (for all sites)***

Take 5 ml nutrient agar tube

Stab the single colony 4-5 times

Incubate at 37ºC for 2-3 days

Seal the vial and store at 4-8º C.

2. ***Storage of samples at AIIMS***

Preservation by Glycerol stock (for AIIMS only)

Add 750 µl of autoclaved BHI media in a 2 ml stocking vial

Then add 250 µl of 60% glycerol in a stocking vial.

Suspend 2-3 loopful of bacteria from culture plates.

Vortex the stocking vial and finally store at -70º C.

Transportation of isolates to reference laboratory

Reference Laboratory – Department of Microbiology, AIIMS, New Delhi

Primary responsibility: **Laboratory technician under supervision of Co-PI (microbiology) of the respective sites**

The cultures should be transported through courier services monthly.

Quality control

A. Internal quality control

The aim of a quality control program would be to monitor the following:

- ✓ Performance of the media and reagents used in the test
- ✓ The precision (repeatability) and accuracy of the susceptibility test procedure
- ✓ The performance of persons

All the participating laboratories would test all media, discs and reagents on the daily basis, to be used for the study.

Sterility: Each batch of medium should be tested for sterility. A plate would be selected at random and incubated at 37ºC for 24-48 hours. The media should be sterile before inoculation. Presence of surface or subsurface colonies warrants discarding of the media.

Growth: The ability of the medium to support the growth is determined by inoculating the medium with a typical stock culture isolate. Diluted inoculum is used for this purpose. Appropriate growth is observed and recorded after incubation of the medium.

Biochemical reactions: Media used for biochemical reactions are tested with strains which give both positive and negative reactions.

A quality control proforma as attached in appendix-4 would be given to all the participating laboratories and on completion need to be sent to the reference laboratory on a fortnightly basis.

B. External quality assessment scheme (EQAS)

1. Reconfirmation

Identification and sensitivity of all the isolates from each centre will be confirmed at AIIMS reference centre through MALDI-TOF and VITEK 2 respectively. One nutrient agar stock will be used for the characterization (i.e. identification and AST). If the details are found similar to that reported by the site, the glycerol stock will be prepared and stored in -80˚C freezer for molecular analysis later. The other nutrient agar stock in this case (which acts as a back-up) will be discarded.

In case there is discrepancy in the characterization of the first nutrient agar stock (as reported by the site), the second nutrient agar stock will be tested.

In case the second nutrient agar stock also do not work, the site will be asked to send the sub-culture (from nutrient agar stock) again stored at the site.

2. Proficiency testing (PT)

The coordinating laboratory at AIIMS, New Delhi would conduct an EQAS for the benefit of participating labs and to ensure uniformity of quality standards. Essentially, lyophilized vials of challenge strain(s) will be sent to the participating laboratory once every quarter, along with details of antimicrobial susceptibility testing procedures to be done and panel of antibiotics to be tested. The laboratories will be required to identify the organism, provide results of susceptibility testing with interpretation of results and special tests done if any. The data obtained will be analyzed at the coordinating laboratory. Each participating laboratory will receive the feedback of their EQAS and recommendations based on their performance.

Annexure 1

Blood culture

Blood culture is the gold standard for diagnosis of sepsis. It should be done in all cases of suspected sepsis **prior to** starting antibiotics.

Blood culture

Equipments needed:

24G/26G disposable needles, 2ml disposable syringe with attached needle, spirit swabs, povidone-iodine swabs, and dry cotton, 1% hypochlorite and puncture-resistant container, and culture medium (20 ml of trypticase soy broth – kept at room temperature)

Precautions:

Wash hands with soap and water for 2 minutes

Steps:

1. Wear sterile gloves prior to the procedure
2. Prepare a circular patch of skin approx. 5-cm in diameter over the proposed puncture site by cleansing the area:
    - i) First with a spirit (70% alcohol) swab; let it dry
    - ii) Clean with 10% Povidone iodine swab; let it dry
    - iii) Clean again with a fresh spirit (70% alcohol) swab

   Note: Always wait till the area becomes dry before cleansing with the next swab; use 70% alcohol procured separately in the project for cleansing the skin (avoid the spirit supplied from the hospital stores)

   Cleanse the area in concentric circles moving outward from the centre
3. Puncture the vein with a sterile 24 or 26G needle. DO NOT break the hub of the needle
4. Once blood starts appearing in the hub, gently withdraw it by using a disposable 2ml syringe and needle (place this needle in the hub of the first needle and withdraw the drop of blood);
5. Withdraw about 1-2 mL of blood

   Note: Check if the culture medium was kept at room temperature before inoculating; also, check for any turbidity in the media before inoculating the blood
6. Remove the leucoplast from the bottle cap; wipe the cap with 70% alcohol swab
7. Insert the needle of the syringe into the cap and slowly inject the blood into the bottle
8. Shake the bottle gently so as to mix the blood and the culture media
9. Label the sample; keep it in the incubator at 37^0C
10. Send to the laboratory within 12-24 hours

Don't:

Do not take blood sample from an existing intravenous or arterial line

Annexure 2

Lumbar puncture (LP)

LP has been recommended as a part of workup of neonatal sepsis since a significant proportion of cases of sepsis (especially culture positive sepsis) are associated with meningitis.

It should be done in *all symptomatic infants* prior to starting antibiotic therapy. If the baby is critically sick and hemodynamically unstable, the procedure should be postponed till the clinical condition stabilizes.

The cerebrospinal fluid (CSF) should be sent for Gram-stain, culture, microscopy (cell count), and biochemical analysis (sugar and protein). Gram stain may provide useful information especially if the infant has received antibiotics before. The characteristics of CSF are unique in the newborn period; the normal values are given in the table below. However, because of the wide range of normal values, CSF protein and sugar values may not be of much help in diagnosing meningitis.

Normal cerebrospinal fluid examination in neonates

CSF components	Normal range
Cells/mm^3	Up to 30 cells
PMN (%)	60%
CSF protein (mg/dl)	Up to 150
CSF/ blood glucose (%)	>60%

(PMN, polymorphonuclear leukocytes; CSF, cerebrospinal fluid)

Lumbar puncture & CSF examination

Equipments needed:

22 to 24G spinal needle or 24G/26G needle, sterile bottles/tubes (at least 3 for collecting CSF), spirit swabs, povidone-iodine swabs, and dry cotton

Precautions:

Obtain the specimen under strict aseptic precautions

Steps:

- Wear sterile gloves prior to the procedure
- Place the neonate in the lateral decubitus position or in the sitting position with legs straightened. One assistant should hold the infant firmly at the shoulders and buttocks. AVOID neck flexion while holding the baby
- Prepare the skin over the puncture site by cleansing the area:
  - i) First with a spirit (70% alcohol) swab; let it dry
  - ii) Clean with 10% Povidone iodine swab; let it dry
  - iii) Clean again with a fresh spirit (70% alcohol) swab

Note: Cleanse the area in vertical motion from above towards the anus

- Cover the cleaned area with sterile towels
- Insert the needle in the midline between the fourth (L4) and fifth (L5) lumbar spinous processes. Use preferably a spinal needle; if not available use ordinary percutaneous needle
- Gradually advance the needle in the direction of the umbilicus; withdraw the stylet (if using spinal needle) to detect the presence of spinal fluid
- Collect CSF in three sterile bottles/tubes; if it is slightly blood stained, collect in one more bottle and discard the first sample; if grossly traumatic (blood-stained), abandon the procedure and repeat after 48 hours

CSF examination

- Inspect the CSF for turbidity and color; it should be clear without any turbidities.
- Use one of these tubes for examination under microscope; the other two samples are sent to the laboratory

Parameter	***Minimum volume needed***	***Procedure***
Cell count - total and differential	0.5 mL	Mix equal amount of CSF and red cell lysing fluid; load one drop of this fluid onto the Newbauer chamber; count the number of cells in the 4 WBC counting chambers. Multiply the number of cells counted by 5 to give the estimated number per mm^3
Glucose and protein	1.0 mL	Send the second sample to the laboratory for glucose and protein estimation; REMEMBER the samples have to be analyzed IMMEDIATELY
Culture	1-2* mL	Send the third tube to the microbiology laboratory

Annexure 3

Burden of multidrug-resistant neonatal sepsis in district hospital settings in India

Statistical Analysis Plan

1. Administrative information

1.1 Title, registration, versions and revisions

**Full study title:**	Burden of multidrug-resistant neonatal sepsis in level-II newborn units of district hospitals in India
**Acronym:**	DH sepsis study
**Local project number:**	N-1965
**SAP version:**	2.0 (January 2022)
**SAP revision history:**	Version 1.0 dated 18 Sep 2019<br>Version 2.0 dated 20 Jan 2022
**SAP revision justification:**	Formatting the draft based on the guidelines for statistical analysis plan<br>Detailed analysis plan for case fatality rates, AMR pattern

1.2 Approved by

We, certify that we read this SAP and approve it as adequate in scope of the main-analyses of the SICS-I.

1.3.1. Principal investigator
Name: Dr M Jeeva Sankar

1.3.2. Statistical consultant
Name: Dr. V. Sreenivas

1.3.3. Co-PI from nodal site
Name: Dr Ramesh Agarwal

1.3.4. Co-PI from nodal site
Name: Dr Kajal Jain

1.3.4. Co-PI from nodal site
Name: Dr Vivek Kumar

Index

Section no	Topic	Page no
1	Introduction	4
2	Objectives	5
3	Methods	6
4	Statistical principles	8
5	Study population	8
6	Analysis	10
7	Discussion and conclusion	16
8	References	17
9	Table and figures	18

2. Introduction

2.1 Background and rationale

Globally, sepsis kills at least half a million neonates every year.[1,2] Often these deaths occur in settings from low- and middle-income countries (LMICs).[1,2] Based on the timing of infection and presumed mode of transmission, neonatal sepsis is categorized into early- and late-onset sepsis: sepsis occurring within first 72 h of life is defined as early-onset sepsis (EOS) whereas that which occurs after 72 h is classified as late-onset sepsis (LOS).[1,2] Generally, EOS infections are considered vertically transmitted (i.e., from mother to neonate – often during intrapartum period) while those causing LOS are considered to be from environmental sources (i.e., horizontal transmission - related to caregiving in hospitals, a type of healthcare associated infection, HAI).[1,2] Traditionally, bacterial pathogens that often cause EOS are different from those causing LOS. For example, *Escherichia coli, group B Streptococci (GBS), Listeria monocytogenes*, and *Enterococcus spp.* are commonly implicated in EOS while *coagulase-negative Staphylococcus spp., Klebsiella spp., Pseudomonas spp., Acinetobacter spp., and Candida spp.* are responsible for LOS.[1–3] Further, EOS differs from LOS with respect to its clinical presentation, antimicrobial resistance (AMR) profiles (of causative pathogens) and outcomes in affected neonates.

This clear distinction between EOS and LOS-causing agents while clearly documented in high-income countries seems to be blurred in LMIC settings. While such observations are reported from tertiary care hospital settings, it is largely unknown if similar conditions, pathogens and antimicrobial resistant profiles persist across neonatal units of secondary and primary care hospitals across India.

Findings from our recent (2011-14) multi-centric Delhi Neonatal Infection Study (DeNIS)[4] suggest that classical LOS-causing pathogens are also associated with EOS.[4] In the DeNIS study, LOS-causing *Acinetobacter spp., Klebsiella spp., Escherichia coli and Staphylococcus aureus* together constituted ~ 65% of EOS burden with Acinetobacter spp., emerging as the predominant one. Additionally, majority (68%) of sepsis cases were EOS (< 72 h), associated with multidrug-resistant (MDR) pathogens, and resulted in high mortality (of up to 59%). This dominance of “nosocomial-type”, LOS-causing bacterial pathogens in

EOS suggests that, in our settings, pathogens causing EOS may be acquired through horizontal transmission perhaps from delivery rooms and/or NICUs within 72 h of birth. As data on neonatal sepsis in secondary hospitals is not available, we will perform an observational study in five neonatal units across secondary/district hospitals and collect high-quality observational data on prevalence of sepsis burden, pathogens causing the burden and their AMR status.

The study will be conducted for about two years in level II special newborn care units (SNCU) of five district hospitals.

2.2 Research question and objectives

2.2.1 Research question

- What is the incidence and incidence density of culture positive sepsis and culture negative sepsis in neonates admitted in the district hospital settings in India?
- What is the prevalence of MDR pathogens (*Acinetobacter* spp., *K pneumoniae*, and *E coli*) in neonates with sepsis in the district hospital settings?
- What is the sepsis-related morbidity and mortality profile in the district hospital settings?

2.2.2 Objectives

The major objectives are to:

1) Determine the burden of sepsis and sepsis-related mortality among neonates admitted in the level-II neonatal units of the in five district hospitals across India
2) Identify the predominant bacterial pathogens that cause early and late-onset neonatal sepsis in these neonates, and
3) Evaluate the antimicrobial resistance profiles of the isolated pathogens in these neonates

The study shall provide data on the following outcomes:

- Incidence of early onset sepsis and late onset sepsis, and culture positive and culture negative sepsis (inborn neonates)
- Prevalence of culture positive and culture negative sepsis (outborn neonates)
- Mortality – all cause as well as sepsis-related mortality
- Profile of organisms isolated

- Antimicrobial resistance pattern
- Details of antibiotic therapy
- Molecular characterization of virulence factors and resistance genes

2.2.3 Scope

This SAP will act as the guiding document for the primary analysis of the project.

3. Study methods

3.1 Study design

This is a prospective cohort study at five level II special newborn care units (SNCU) of district hospitals under supervision of tertiary sites (depicted in the following table):

Name of District Hospital	Number of beds	Interventions available	Annual admissions
Government Headquarters Hospital, Cuddalore, Tamil Nadu	20	a) CRP testing<br>b) Hemogram<br>c) CPAP<br>d) Ventilator<br>e) Microbiology lab	5789 live births, 1204 SNCU admissions (836 inborn +368 outborn) [2017 data] and 586 live births, 996 SNCU admissions (655 inborn +341 outborn) [**2018 data**]
District Hospital Mahasamund, Chhattisgarh	12	Facility for CRP (Qualitative) and hemogram testing	1709 live births,902 SNCU admissions (294 inborn +608 outborn) [2017 data] and 1821 live births,1100 SNCU admissions (341 inborn +759 outborn) [**2018 data]**
Regional Hospital Una, Himachal Pradesh	8	Facility for CRP and hemogram testing	3457 live births,724 SNCU admissions (617 inborn +107 outborn) [**2017 data**] and 3919 live births, 755 SNCU admissions (476 inborn +279 outborn) [**2018 data**]
Civil Hospital, Sivasagar, Assam	12	Facility for CRP and hemogram testing	1035 SNCU admissions (**2016 data**)
Government Nahata Hospital, Balotra, Barmer Rajasthan	12	Facility for CRP and hemogram testing	Around 60 neonates admitted every month with 60% being outborn

All the neonates (inborn and outborn) admitted in the study hospitals will be enrolled and tracked for the occurrence of sepsis. In neonates with suspected sepsis, clinical work up, isolation of pathogens and antibiotic susceptibility test will be done as per the defined standard operating procedures. The neonates will be tracked for final outcome until death or 28 days of age.

The study proposal was approved by the IEC and EC at the 5 district hospitals as well as the five accompanying tertiary care sites where the lab work will be conducted, in addition to the nodal centre at AIIMS, New Delhi.

3.2 Sample size

Among the various outcomes of the study, we chose the prevalence of multi-drug resistance (MDR) among the four common pathogens namely, Acinetobacter spp., K pneumoniae, S aureus, and E coli to estimate the required sample size. In the DeNIS study, the prevalence of MDR varied from 38% to 82% for the three common Gram-negative pathogens (Acinetobacter spp., K pneumoniae, and E coli) and 38% to 61% for the two common Gram-positive pathogens (S aureus and CoNS).[4] Assuming the prevalence of MDR to be 30%, an absolute precision of 7%, and a type 1 error rate of 0.05, we have to enroll 165 neonates with culture-positive sepsis. If the culture-positivity rate is 4% of all SNCU admissions, we would have to enroll about 4150 neonates in total. The expected number of admissions in the five proposed sites is around 2000 to 2100 per year.

3.3 Timing of final analysis

The original timeline for study completion was September 2021, however due to CoVID, there has been delay in enrolments. As per revised estimates, the study enrolment is expected to be completed by December 2022. The final analysis shall be conducted after the last infant enrolled in study completes 28 days of life.

3.4 Timing of outcome assessment

All neonates will be followed till 28 days of life for the final outcome assessment.

S. No.	Outcomes	Timing of assessment
1.	Incidence of early onset sepsis	Till 72 hours of life
2.	Incidence of late onset sepsis	After 72 hours and till 28 days of life
3.	Mortality<br>In-hospital mortality<br>28-day mortality	 Till hospital discharge<br>Till 28th day of life
4	Mortality<br>All-cause mortality<br><br>Sepsis related mortality	 Death within 28 days, irrespective of the cause of death<br><br>Death within 28 days, attributed to sepsis

4. Statistical principles

4.1 Confidence intervals and P-values

Data will be summarized using mean and standard deviation for continuous variables and percentages for categorical variables. Skewness of continuous data will be assessed by examining the histogram and by using tests like Shapiro wilk test; skewed data will be presented as median (IQR). The primary outcome variables – incidence of sepsis – shall be expressed as proportions along with 95% confidence intervals. The confidence interval shall be adjusted for clustering of neonates within the sites using the svyciprop function from the 'survey' package in R. All significance level shall be set at p value 0.05.

4.2 Description and rationale for any ***adjustment for multiplicity*** and, if so, detailing how the type 1 error is to be controlled:

Not applicable

4.3 Adherence and protocol deviations

4.3.1 Definitions of protocol deviations for the trial

Protocol deviations are defined when the activities on a study diverge from the local institutional review board-approved protocol, however without significant consequences.[5] Being an observational study, we do not expect any protocol deviations.

4.3.2 Description of which protocol deviations will be summarized

Not applicable

5. Study population

5.1 Screening data

We shall collect data for all eligible neonates who were included in the study. We shall document the reasons for exclusions in neonates who are excluded based on pre-specified exclusion criteria. But we don't expect any major exclusions in this observational study.

5.2 Eligibility

5.2.1 Inclusion criteria

All neonates (inborn and outborn) admitted in the SNCUs of the district hospitals; postnatal age less than or equal to 28 days

5.2.2 Exclusion criteria

Parents not willing to participate in the study

5.3 Recruitment

All eligible neonates admitted in the SNCUs of the district hospitals for any indication during the study period will be tracked by the research staff and enrolled after taking informed consent from parents/ legally acceptable representative (LAR). The flow has been shown in figure 1.

5.4 Withdrawal/follow-up

5.4.1 Level and timing of withdrawal

There are no expected dropouts for the in-hospital outcome variables, but a proportion of parents may not be reachable for outcome assessment of the neonate at day 28 of life after being discharged/ referred or LAMA.

5.4.2 Reasons and details of withdrawal

Reasons for withdrawal or lost to follow-up will be reported in the manuscript and/or flow diagram. Our observational study consists of tracking and follow up of neonates till 28 days of life. They will be contacted telephonically at 28th day of life if they had left against medical advice, were discharged or referred before the 28th postnatal day.

5.5 Baseline patient characteristics

5.5.1 Collected baseline patient characteristics

Maternal socio-demographic characteristics, perinatal details and neonatal demographic characteristics shall be recorded in a pre-designed case record form (CRF). The reasons for suspecting sepsis, onset of sepsis, and final labelling of sepsis shall also be recorded in the CRF.

5.5.2 Descriptive summarization of baseline patient characteristics

We shall list the maternal and neonatal characteristics in a baseline table. Statistical analysis shall be done with Stata 15.1 (StataCorp, College Station, TX) and R version 3.6.3 (The R Foundation for Statistical Computing). Continuous variables shall be presented as mean with standard deviation (SD), when normally distributed or as median with interquartile range, if skewed. Categorical data shall be presented as proportions.

6. Analysis

6.1 Outcome definitions

Standard definitions that will be used in the study are as follows:

**Suspected sepsis**	Age of the neonate ≤28 days<br>AND<br>Presence of any one of the risk factors/ clinical symptoms/signs from the following list, for which the neonate has no other known or reliable explanation (*to be verified independently by a neonatologist from the corresponding tertiary site later)*:<br>***Perinatal risk factors***<br>1. Foul-smelling liquor<br>2. At least two of the following: (i) spontaneous prematurity and preterm pre-labor rupture of membranes (PPROM; irrespective of duration of rupture of membranes) (ii) Febrile illness in the mother with suspected bacterial infection warranting start of antibiotics by attending clinician in the week prior to delivery (iii) rupture of membranes >24 hours (iv) unclean vaginal examination(s) during labor<br>***Clinical symptoms/signs***<br>3. No movement or movement only when stimulated<br>4. Refusal to feed<br>5. Severe chest in-drawing or increased oxygen requirement or need for respiratory support<br>6. Grunting<br>7. New onset apnea or increased severity or frequency of apnea in a baby who already is having apneas<br>8. Cyanosis or desaturation needing oxygen therapy or respiratory support (or increase in oxygen requirement/deterioration in a baby who is already receiving oxygen therapy)<br>9. Fever or hypothermia (>37·5˚C or <36·5˚C)<br>10. Tachycardia or episodes of bradycardia (>180/min or <100/min)<br>11. Capillary refill time (CRT) > 3 sec<br>12. Mottled skin or other evidence of shock<br>13. Erythema in the skin around umbilical stump (extending to >1 cm)<br>14. Lethargy or drowsiness<br>15. Convulsions<br>16. Abnormal posturing<br>17. Hypotonia or floppiness<br>18. Bulging fontanelle<br>19. Vomiting or abdominal distension<br>20. Bleeding<br>21. Sclerema
**Culture-positive sepsis**	Neonate with 'suspected sepsis' (see above) AND isolation of a recognized pathogen* from blood, cerebrospinal fluid, or other body fluids.<br>If *CONS**, it should be cultured from one or more blood samples drawn on separate occasions and organism cultured is not related to an infection at another site.
**Culture-negative**	Neonate with 'suspected sepsis' AND negative blood culture (or blood culture

**sepsis**	deemed to have grown a commensal) AND (clinical course consistent with sepsis or the neonate received at least 5 days of antibiotics)
**Early-onset sepsis**	Occurrence (i.e., time of onset of symptoms) of culture-positive or culture-negative sepsis at or before 72 h of life
**Late-onset sepsis**	Occurrence (i.e., time of onset of symptoms) of culture-positive or culture-negative sepsis after 72 h of life
**Meningitis**	Positive cerebrospinal fluid culture, Gram staining, or neutrophilic leukocytosis, with or without low glucose (<50% of plasma glucose level) and high protein content<br>(Normal range of CSF components: cells - up to 30 cells/mm^3; polymorphonuclear leukocytes (PMN) - 60%; protein: up to 150 mg/dL; CSF/blood glucose >60%
**Systemic fungal infection**	Blood culture, CSF, or suprapubic urine positive for yeasts or presence of budding yeast/hyphae AND<br>physician institutes appropriate therapy for fungal infection
**New episode of sepsis**	When the neonate become symptomatic after 48 hours of stopping appropriate antibiotic therapy, or if a new organism is cultured, with fresh clinical deterioration during an ongoing episode.
**Case-fatality rate**	Death due to sepsis within 28 days of life or 21 days of suspicion, whichever is earlier. For each death, single-most underlying cause of death was assigned by the investigator based on the criteria outlined in National Neonatal Perinatal database (NNPD)

6.1.1 Primary outcome

The primary outcome of the study is to determine the incidence of culture positive sepsis in enrolled neonates at district hospitals of India

6.1.2 Secondary outcomes

a) Incidence of early-onset sepsis (EOS) and late-onset sepsis (LOS)
b) Mortality among neonates with sepsis (overall, culture-positive and culture-negative) as per site
c) Pathogen profile in early- vs late-onset sepsis and as per sites
d) Antimicrobial resistance pattern of major gram-negative and gram-positive pathogens
e) Determine the prevalence of MDR pathogens- *Acinetobacter* spp., *K pneumoniae*, and *E coli; which have been* commonly implicated in tertiary care settings
f) Case fatality rate in neonates with culture-positive sepsis caused by common pathogens, segregated by their antimicrobial resistance pattern
g) Molecular characterization of virulence factors and resistance genes of common pathogens
h) Identify maternal and perinatal risk factors responsible for developing culture positive and culture negative sepsis in neonates
i) Readmissions: frequency, reason and time to re-admission
j) Usage of antibiotics at the sites: number of antibiotics per neonate; type and duration of antibiotics used; and number of neonates with change in antibiotic therapy and reason for change
k) Time to occurrence of clinical symptom and time to resolution after therapy: Clinical signs of each neonate are observed daily. These observations will be characterized and tabulated. The response time shall be correlated with the antibiotics being administered
l) Determine the diagnostic value of complete blood counts (CBC), absolute neutrophil counts (ANC), and semi-quantitative CRP to diagnose culture positive sepsis

6.1.3 Measurement and calculation of outcomes

Clinical and microbiology data will be collected in the respective forms. The data would be checked for by the assigned monitors (during their visit to the site) and verified with

source documents. Forms after being cross-checked by DH investigator and the corresponding mentoring tertiary site investigator will be entered in an electronic data capture platform by the data entry operators/research nurses (on-site) as per the eCRF filling guidelines.

The data recorded in Web/Forms will be stored in a secure electronic database accessible only to authorized personnel. Investigators will be asked to verify all recorded data for quality control/validation. The data will also be checked with respect to range, internal consistency, missing or extreme values, and errors. Inconsistencies or missing data will be resolved in discussion with the site investigators.

The primary outcome variable – incidence of sepsis – shall be expressed as proportions along with 95% confidence intervals. The confidence interval shall be adjusted for clustering of neonates within the sites using the svyciprop function from the 'survey' package in R. The incidence rate of sepsis shall be calculated as the number of episodes of sepsis per 1000 patient-days.

Hazard ratios: The Cox proportional hazards model will be used to estimate the hazard ratios and 95% CIs for all-cause neonatal mortality within 28 days for neonates with culture-positive sepsis, culture-negative sepsis, and clinically suspected sepsis but not labelled as either culture-positive or culture-negative sepsis, compared to neonates not suspected to have sepsis. The model will be adjusted for potential confounding factors, including birth weight, gestational age, birth asphyxia, and the presence of major congenital malformations. Additionally, to account for the clustering effect of the study site a robust variance estimator using the cluster option from package 'survival' in R will be used. Kaplan-Meier survival curves will be constructed to visualize the differences in survival probabilities among the four sepsis categories. The survival curves will be generated using the 'surfeit' function and the 'ggsurvplot' package. The log-rank test p-value will be used to assess the overall significance of the survival differences among the groups.

Pathogen profile: The number of pathogens isolated from culture-positive neonates will be expressed as frequencies; if two pathogens are isolated from a single sepsis episode in a neonate, both will be included while counting the number of pathogens.

Antimicrobial resistance (AMR): AMR is generally reported as susceptible,

intermediate, resistant, or not tested for individual antibiotics; the 'intermediate' category will be merged with the 'resistant' category for the study. Additionally, the Gram-negative pathogens will be classified further based on their resistance to the following antibiotic classes: 3rd generation cephalosporins (any one of cefotaxime, ceftriaxone, or ceftazidime); carbapenems (any of imipenem, meropenem or ertapenem); aminoglycosides (any of gentamicin, amikacin, or netilmicin); fluoroquinolones (ciprofloxacin); β-lactam/β-lactamase inhibitors (BLBLI; any of amoxicillin-clavulanic acid, piperacillin-tazobactam, or cefoperazone-sulbactam); and polymyxins (colistin). Multidrug resistance was defined as resistance to one or more agents in at least three of the above antibiotic classes.

Case-fatality rates (CFR): Case-fatality rate will be calculated by dividing the number of deaths due to sepsis within 28 days of life or 21 days of suspicion of sepsis, whichever is earlier, by the number of neonates in the respective sepsis category (i.e. culture positive sepsis or culture negative sepsis). In case of a neonates having episodes of both culture-positive and culture-negative sepsis, the neonate will be included in the culture positive category.

A subgroup analysis of CFR will be done among Gram-positive and Gram-negative organisms. It will be calculated by dividing the number of deaths due to sepsis within 28 days of life or 21 days of suspicion of sepsis, whichever is earlier, by the number of neonates with culture positive for Gram-positive and Gram-negative organisms respectively. In case of a neonates having episodes of both Gram-positive and Gram-negative organisms, the neonate will be included in the Gram-negative category.

Sub-group analysis: The incidence, pathogen profile, antimicrobial resistance and case fatality rates shall be calculated in the following subgroups- site, place of birth, gestation, birth weight, onset of sepsis and sepsis categories.

Exploratory analysis: In addition to the descriptive outcomes, as an exploratory analysis, we shall also identify the maternal and perinatal risk factors of culture positive and culture negative sepsis by using standard bivariate and multivariate logistic regression analyses. In multivariate model(s), we shall use the following pre-specified clinical variables known to be associated with neonatal sepsis: birth weight, gestation at birth, mode of delivery, major malformation, antenatal steroid coverage (in <34 weeks), meconium stained liquor, foul smelling liquor, unclean vaginal examination, rupture of

membranes>18 hours, maternal antibiotics within 7 days of delivery, whether any medication applied to the cord, requirement of resuscitation at birth and administration of pre-lacteal feeds. In addition, we will include those baseline variables which are statistically significant (P <0.1) in bivariate analyses and are clinically relevant as independent variables. Risk factors with prevalence of less than 1% in the overall cohort will not be included in multivariable analysis. Culture-positive and culture- negative sepsis will act as the dependent variables. A p-value of <0.05 for the model will be taken as significant. The discriminatory power of the model will be assessed by area under the receiver operating characteristic curve (AUC) along with its 95% CI.

Calculations will be performed as per the statistical tests and methods mentioned above. Dummy tables have been presented in the annexure attached with the analysis plan.

6.2 Sensitivity and subgroup analyses

CoNS are known skin contaminants but have also been shown to be pathogenic in neonates. We will perform a sensitivity analysis for the primary outcome (estimating the incidence of culture positive sepsis) after excluding CoNS as pathogens.

We do not intend to adjust the p-values for the pre-specified multiple subgroup analyses based on the sites, birthweight categories, gestational age categories, and fetal growth (small-for-gestational age vs. appropriate-for-gestational age) categories.

6.3 Missing data

6.3.1 Reasons for missing data: Neonates with missing data for some variables shall be excluded from the denominator.

6.3.2 Imputation method: No imputation will be done for the missing data

6.4 Additional analyses

We will maintain log of screened infants and the source documents for each enrolled neonate. The following points shall be observed:

- The data will be recorded in a source file and thereafter entered electronically into a central database with all entries and modifications in an audit trail mode.
- Access to data will be granted by administrator after appropriate and documented training.
- Automated validation checks for data discrepancies

On-site and remote monitoring i.e. review of case report form, medical records and other study records to verify data quality and integrity shall be performed by the monitor(s) at regular intervals (as defined in the Clinical Operations Plan/Monitoring Plan) throughout the study period.

6.5 Statistical software

Statistical analysis shall be done with Stata 15.1 (StataCorp, College Station, TX) and R version 3.6.3 (The R Foundation for Statistical Computing).

7. Discussion

The overall aim of the DH sepsis study is to determine the incidence of culture positive and culture negative sepsis. It will also shed light on the prevalent pathogens and their antibiotic susceptibility patterns in the district hospital settings in India. This will help in framing antibiotic policies as well antibiotic stewardship efforts in district hospitals. As a secondary analysis we will also try to find clinical risk factors which can predict the probability of occurrence of sepsis. It will test only the discriminatory ability of the model and will require validation in future studies from a similar cohort. We drafted this SAP to avoid outcome reporting bias and data-driven results.

8. Conclusion

This SAP presents the principles of analysis of the DH sepsis cohort and discusses its major methodologic and statistical concerns. We hope that the results of the project will be as transparent and robust as possible, so that we minimize the risk of outcome reporting bias and data-driven results.

9. References:

1. Shane AL, Sánchez PJ, Stoll BJ. Neonatal sepsis. Lancet Lond Engl. 2017 Oct 14;390(10104):1770–80.

2. Shane AL, Stoll BJ. Neonatal sepsis: progress towards improved outcomes. J Infect. 2014 Jan;68 Suppl 1:S24-32.

3. Zea-Vera A, Ochoa TJ. Challenges in the diagnosis and management of neonatal sepsis. J Trop Pediatr. 2015 Feb;61(1):1–13.

4. Investigators of the Delhi Neonatal Infection Study (DeNIS) collaboration. Characterisation and antimicrobial resistance of sepsis pathogens in neonates born in tertiary care centres in Delhi, India: a cohort study. Lancet Glob Health. 2016 Oct;4(10):e752-760.

5. Bhatt A. Protocol deviation and violation. Perspect Clin Res. 2012;3(3):117.

6. Sievert DM, Ricks P, Edwards JR, Schneider A, Patel J, Srinivasan A, et al. Antimicrobial-resistant pathogens associated with healthcare-associated infections: summary of data reported to the National Healthcare Safety Network at the Centers for Disease Control and Prevention, 2009-2010. Infect Control Hosp Epidemiol. 2013 Jan;34(1):1–14.

Annexures

Figures

Figure 1: Study flow

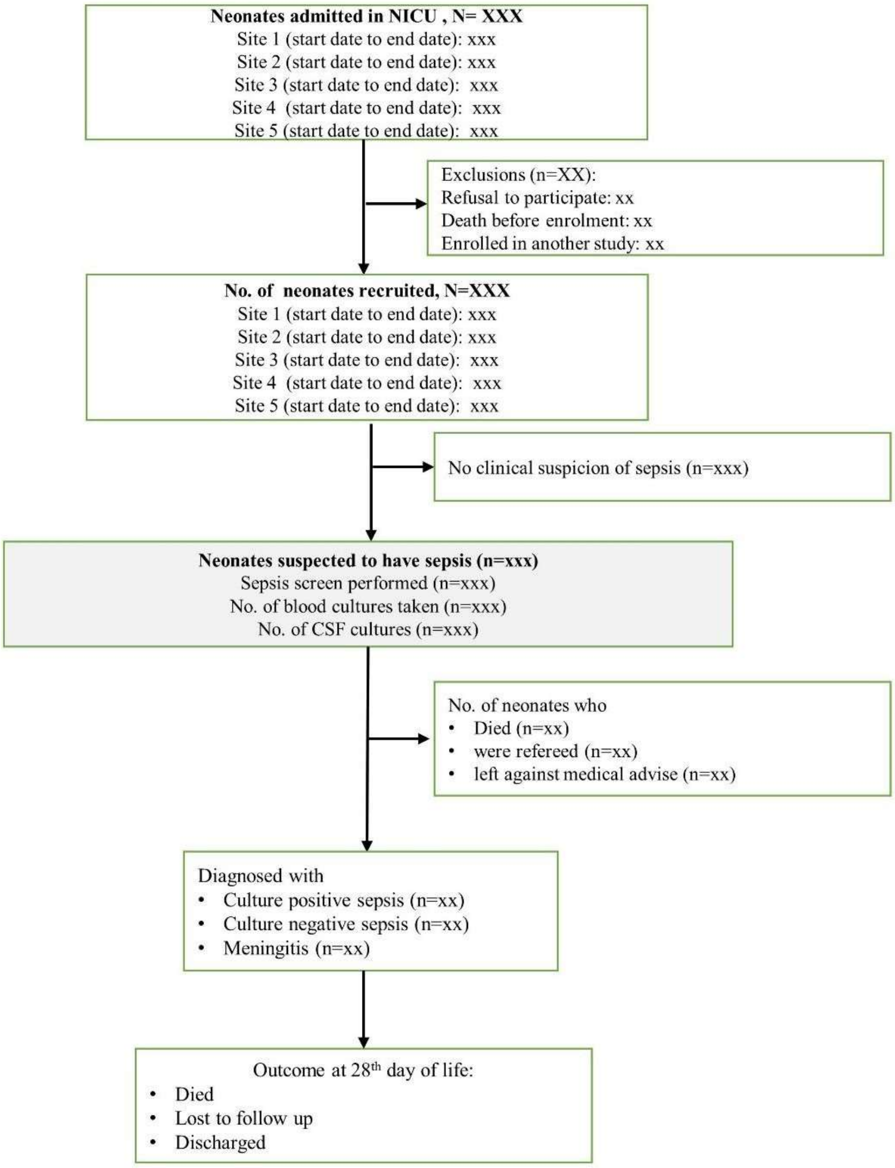

Dummy tables

i) Enrolment:

- Overall
- By centre

Table 1: Number of neonates enrolled at sites

Site	Number of neonates enrolled during study period of ___to ____	Number of neonates with complete follow-up during study period of
Cuddalore		
Mahasamund		
Nahata		
Sivasagar		
Una		
**Total**		

ii) Baseline characteristics

Categorical data will be tabulated: overall and as per centre. Continuous data will be summarized as mean (standard deviation) or median (interquartile range): overall and by centre.

Table 2: Baseline characteristics

**Neonatal characteristics**						
	**Overall**	**Site 1**	**Site 2**	**Site 3**	**Site 4**	**Site 5**
Gestation (wk)<br>• N (%)<br>• mean, SD<br>• median, range						
Gestation groups (wk)<br>• 26-27<br>• 28-32<br>• 33-37<br>• >37						
Birth weight (g)<br>Mean ± SD<br>Median (Range)						
Birth weight groups (g)<br>• <750<br>• 750-999<br>• 1000-1249<br>• 1250-1499<br>• 1500-1999<br>• 2000-2999<br>• ≥3000						
Fetal growth category<br>• SGA<br>• LGA						
Females						
Multiplicity						

• Single<br>• Twin<br>• Triplet						
Babies cried at birth						
Mean age at admission in SNCU						
Place of delivery<br>• Home<br>• Same district hospital<br>• Other hospital						
Malformations present						
**Maternal Characteristics**						
Age of mother<br>• N (%)<br>• mean, SD<br>• median, range						
Education of mother-completed years<br>• N (%)<br>• mean, SD<br>• median, range						
Education of father-completed years<br>• N (%)<br>• mean, SD<br>• median, range						
Mean family income						
Antenatal steroid<br>• N<br>• Multiple course<br>• Single course<br>• Incomplete<br>• None<br>• Not applicable						
Mean duration of rupture of membrane						
Mode of delivery<br>• Emergency cesarean<br>• Elective caesarean section<br>• Vaginal-Instrumental<br>• Vaginal-Induced<br>• Vaginal-spontaneous<br>• Unknown						

Meconium stained liquor						
Antibiotics in last 7 days before delivery						
Mean duration of antibiotics before labor, if administered						
Most common antibiotics received by mothers before delivery						
Mothers with unclean (ungloved) intrapartum vaginal examination during labor						
Was the umbilical cord cut with a sterile instrument?						
Was anything applied to the cord?						
If **YES**, what was applied?						
Pre-lacteal feeds given to the baby						

iii) Previous hospitalization and antibiotic history for extramural neonates

Table 3: Previous hospitalization and antibiotic history for extramural neonates

Delivery attended by:<br>• Doctor / Nurse<br>• Skilled midwife<br>• Traditional birth attendant<br>• Family member<br>• Others	
Number of babies who received any antibiotics on OPD basis (oral or injectables)?	
Number of babies who received any previous hospitalization	
Mean duration of previous hospitalization	
Type of hospital where baby was admitted<br>• Primary health facility<br>• District hospital<br>• Tertiary hospital<br>• Small private nursing home<br>• Large private hospital<br>• Others	
Number of babies who received antibiotics	
Most common antibiotics received by the baby in previous hospitalization	
Duration of antibiotics received *(days)* in previous hospitalization	
Number of re-admissions	

iv) Details of episodes of sepsis among neonates: suspected sepsis, culture-positive sepsis and culture-negative sepsis

- Frequency: site-wise, place of birth
- postnatal age (days) of suspecting sepsis
- most common three signs/ symptoms
- Mean cell counts

Table 4: Details of episodes of sepsis among neonates

	Suspected sepsis	Culture positive sepsis	Culture negative sepsis
N			
Mean postnatal age (days) of suspecting sepsis			
Most common three signs/ symptoms			
Mean WBC count (cells/L)			
Mean ANC (cells/L)			
CRP>6 mg/L			

v) Primary outcome:

Incidence of early onset sepsis and late onset sepsis, and culture positive and culture negative sepsis (inborn and outborn neonates) will be calculated by dividing the frequency/number of neonates with culture-positive sepsis with total number of enrolled neonates. The 95% confidence interval around that will be calculated.

Table 5: Incidence of sepsis among neonates

	**Incidence** (frequency/ SNCU admissions)	**95% confidence interval** (adjusted for clustering within site)
Culture-positive sepsis		
Culture-negative sepsis		
Early-onset		
Late-onset		

Table 5A: Incidence and incidence density of sepsis among inborn neonates

	**Incidence** (frequency/ SNCU inborn admissions)	**95% confidence interval** (adjusted for clustering within site)
Culture-positive sepsis		
Culture-negative sepsis		
Early-onset		
Late-onset		

Table 5B: Prevalence of sepsis among outborn neonates

	**Prevalence**<br>(frequency/ SNCU outborn admissions)
Culture-positive sepsis	
Culture-negative sepsis	
Early-onset	
Late-onset	

vi) **Mortality** – all cause as well as sepsis-related mortality: Primary and secondary causes of death will be described.

Mortality of neonates (all-cause and with sepsis) at 28 days of age will be calculated as percentage (overall, as per site, place of delivery, readmissions, as per culture-positivity and pathogen in culture-positive sepsis)

1. Site-wise details of sepsis episodes among neonates
2. Site-wise details of neonates with sepsis
3. Mortality among neonates with sepsis (overall, culture-positive and culture-negative) as per site
4. Pathogen profile in early- vs late-onset sepsis, as per site
5. Antimicrobial resistance pattern of major gram-negative and gram-positive pathogens
6. Antimicrobial resistance pattern of major gram-positive pathogens
7. Case fatality rate among common pathogens by their antimicrobial resistance pattern

Table 6: Site-wise details of sepsis episodes among neonates

	Culture-positive sepsis episodes	Culture-negative sepsis episodes
Overall		
Site 1		
Site 2		
Site 3		
Site 4		
Site 5		

Table 7: Site-wise details of neonates with sepsis (as per place of birth)

	Enrolled neonates	Neonates with culture-positive sepsis	Neonates with culture-negative sepsis
Overall<br>• Inborn<br>• Outborn			
Site 1<br>• Inborn<br>• Outborn			
Site 2<br>• Inborn<br>• Outborn			
Site 3<br>• Inborn<br>• Outborn			
Site 4<br>• Inborn			

• Outborn			
Site 5<br>• Inborn<br>• Outborn			

Table 8: Site-wise details of neonates with sepsis (as per time of onset of sepsis)

	Enrolled neonates	Neonates with culture-positive sepsis	Neonates with culture-negative sepsis
Overall<br>• early<br>• Late			
Site 1<br>• Early<br>• Late			
Site 2<br>• Early<br>• Late			
Site 3<br>• Early<br>• Late			
Site 4<br>• Early<br>• Late			
Site 5<br>• Early<br>• Late			

Table 9: Case-fatality rate among neonates with sepsis

	Deaths/ neonates with culture-positive sepsis	Deaths / Neonates with culture-negative sepsis
Overall<br>• Inborn<br>• Outborn		
Site 1<br>• Inborn<br>• Outborn		
Site 2<br>• Inborn<br>• Outborn		
Site 3<br>• Inborn<br>• Outborn		
Site 4<br>• Inborn<br>• Outborn		
Site 5<br>• Inborn<br>• Outborn		

Table 10: Pathogen profile in early- vs late-onset sepsis

Pathogen	Early-onset sepsis		Late-onset sepsis	
	Inborn	Outborn	Inborn	Outborn

Table 11: Pathogen profile by site

Pathogen	Overall	Site 1	Site 2	Site 3	Site 4	Site 5

Table 12: Antimicrobial resistance pattern of major gram-negative pathogens

Antibiotic	*Acinetobacter spp*	*Klebsiella spp*	*Enterobacter spp*	*Pseudomonas spp*	*E coli*
Amoxicillin					
Amikacin					
Cefotaxime					

Table 13: Antimicrobial resistance pattern of major gram-positive pathogens

Antibiotic	*Coagulase-negative staphylococci*	*Staphylococcus aureus*	*Enterococcus spp*

Table 14: Case fatality rate among common pathogens by their antimicrobial resistance pattern

Pathogen	CFR in culture positive sepsis due to resistant pathogens /Number of resistant pathogens	CFR in culture positive sepsis due to sensitive pathogens / Number of sensitive pathogens
